# Differentiable graph clustering with structural grouping for single-cell RNA-seq data

**DOI:** 10.1093/bioinformatics/btaf347

**Published:** 2025-06-13

**Authors:** Xiaoqiang Yan, Shike Du, Quan Zou, Zhen Tian

**Affiliations:** School of Computer and Artificial Intelligence, Zhengzhou University, Zhengzhou 450000, China; School of Computer and Artificial Intelligence, Zhengzhou University, Zhengzhou 450000, China; Yangtze Delta Region Institute (Quzhou), University of Electronic Science and Technology of China, Quzhou 324000, China; School of Computer and Artificial Intelligence, Zhengzhou University, Zhengzhou 450000, China; Yangtze Delta Region Institute (Quzhou), University of Electronic Science and Technology of China, Quzhou 324000, China

## Abstract

**Motivation:**

Clustering cells into subpopulations is one of the most crucial tasks in single-cell RNA sequencing (scRNA-seq) data analysis, which provides support for biological research at cellular level. With the development of graph neural networks, deep graph clustering approaches have achieved excellent performance by modeling the topological relationships between cells. However, existing approaches rely on cell node and its neighbors to obtain the cell feature representation, which ignore the graph cluster structure hidden in scRNA-seq data. Besides, how to bridge the heterogeneous gap between cell node feature and its structural information remains a highly challenging problem.

**Results:**

Here, we propose a novel differentiable graph clustering with structural grouping (DGCSG) for scRNA-seq data, which incorporates graph cluster information into deep graph clustering model by designing a differentiable clustering mechanism to learn clustering-friendly representation. Firstly, an interactive module is devised to dynamically transfer node representations learned by autoencoder (AE) to graph attention autoencoder (GATE) in layer-by-layer manner. Then, to characterize graph cluster information, a differentiable clustering mechanism is proposed to transform K-way normalized cuts from a discrete optimization problem into differentiable learning objective through spectral relaxation, which jointly optimizes the GATE by allocating more attention scores to nodes in the same graph cluster. Finally, a decoupled self-supervised optimization is proposed, which guides the representation learning of AE and GATE in the interactive module. Extensive evaluations on 14 scRNA-seq benchmarks verify the superiority of DGCSG compared with state-of-the-art baselines.

**Availability and implementation:**

The code associated with this work is available on GitHub (https://github.com/Xiaoqiang-Yan/DGCSG).

## 1 Introduction

Driven by innovations in high-throughput sequencing technology, a massive amount of single-cell RNA-sequencing (scRNA-seq) data is generated, which provides support for measuring transcriptional gene expression at cellular resolution. Based on the high-throughput sequencing information, cell type discovery has become the basis for various complex biological tasks, such as drug discovery (Tan *et al.* 2024), disease diagnosis (Khan *et al.* 2024), and cancer research (Marot-Lassauzaie *et al.* 2024, Tirosh and Suva 2024). However, technical and biological factors cause scRNA-seq data to present complex characteristics such as high dimensionality, significant sparsity, and substantial noise (Angerer *et al.* 2017). These characteristics make it challenging to accurately discover cell types from large-scale data.

To address the aforementioned difficulties of cell type discovery, various traditional clustering approaches have been proposed, which aim to partition unlabeled scRNA-seq data into biologically meaningful cell clusters according to gene expression patterns. For example, Wang *et al.* (2017) learned cell similarity measure through multikernel learning. Kiselev *et al.* (2017) and Žurauskienė and Yau (2016) projected high-dimensional gene expression data to a low-dimensional feature space based on principal component analysis. Xu *et al.* (2023) provided a single-cell clustering tool to identify important genes through random forests. However, these traditional clustering approaches encounter challenges in precisely modeling the nonlinear characteristics of scRNA-seq data since they leverage objective functions based on linear embedding.

In recent years, lots of deep scRNA-seq clustering approaches have been designed to learn discriminative representation from scRNA-seq data, in which one of the most popular deep clustering frameworks is autoencoders (AEs). For example, as a pioneer work, Eraslan *et al.* (2019) designed a deep count autoencoder (DCA) model, which adapts a typical AE to denoise scRNA-seq data. Inspired by DCA, Tian *et al.* (2019) developed a scDeepCluster model to learn cell features through zero-inflated negative binomial (ZINB) loss. Li *et al.* (2020) utilized stacked AE to iteratively learn cell representations and cluster assignments for scRNA-seq data. Besides, some methods incorporate AE with traditional clustering models together to enhance the scRNA-seq clustering performance. For example, Chen *et al.* (2020) characterized scRNA-seq data by utilizing a denoising AE, and then clustered the learned cell representations with a traditional K-means algorithm. Recently, contrastive learning-based methods are proposed to learn cell representations by constructing positive and negative sample pairs, which minimize the similarity of negative pairs while maximizing that of positive pairs in the representation space. For example, Ciortan and Defrance (2021) employed a self-supervised contrastive learning framework to learn the augmented data representation and then clustered the representations learned by the model. Wan *et al.* (2022) combined a mask estimation task with a contrastive learning framework to facilitate AE learning clustering-friendly representations. However, these aforementioned AE-based clustering approaches and their variants ignore the typological information between different cells, thereby hardly capturing cell–cell relationships.

To leverage cellular typological information and improve clustering accuracy, some researchers resort graph neural networks (GNNs) to model the relationships among cells. GNN-based clustering approaches first construct cell–cell graph and then learn cell representations from the whole graph. For example, Wang *et al.* (2021) utilized GNN to aggregate cell–cell relationships and reduce the impact of excessive dropout events on downstream analysis. Liu *et al.* (2024a) designed a dual-channel network including AE and GNN to fuse node and structural features. Yu *et al.* (2022) learned latent representations by combining ZINB loss and topologically adaptive graph convolutional neural network. Although existing GNN-based approaches have made significant progress in scRNA-seq clustering, they still have the following limitations: (i) The high dimensionality, significant sparsity and substantial noise of scRNA-seq data pose challenges to constructing a reliable graph and accurately characterizing cell relationships. In other words, inaccurate structural information undermines clustering performance because the information-sharing pattern is disrupted. (ii) GNNs often convey information among adjacent cells anonymously. Given the inherent noise in scRNA-seq data, numerous edges in the constructed graph are misconnected. Therefore, it is necessary to employ an attention mechanism to accurately capture graph structure information.

**Figure 1.**
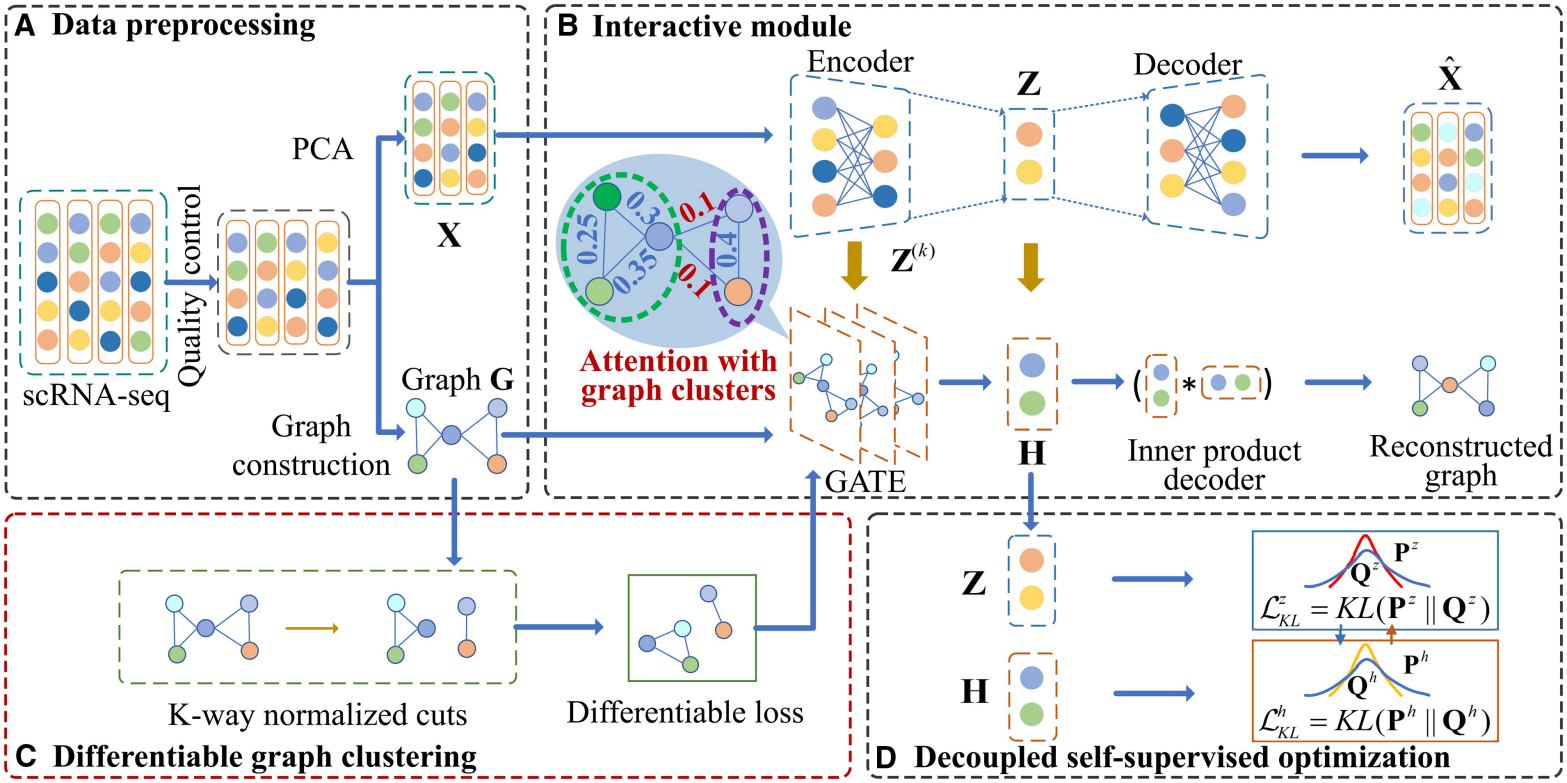
The model architecture of DGCSG. DGCSG is composed of four modules: (A) data preprocessing, (B) interactive module, (C) differentiable graph clustering, (D) decoupled self-supervised optimization. Firstly, the data preprocessing module generates a gene expression matrix X and a cell–cell graph G. Next, X and G are input to the interactive module for representation learning, and the learned representations are dynamically fused layer by layer. Meanwhile, the differentiable graph clustering module utilizes the cell–cell graph to learn graph cluster information and assists GATE in assigning higher attention scores to nodes within the same cluster through the differentiable loss. Finally, the decoupled self-supervised optimization module is devised to guide the learning of representations Z and H.

The attention mechanism calculates the importance of node relationships through a learnable attention score, assigning different weights to the links between nodes in the constructed graph, thus capturing the relationships between nodes more accurately. For example, Cheng and Ma (2022) achieved promising results on scRNA-seq clustering by designing a graph attention network. However, existing models based on attention mechanism still have the following shortcomings: (i) in cell–cell graphs, there often exists the heterogeneous gap between the feature representation of cellular node and their structural information, which might interfere clustering performance on scRNA-seq data (the difference between the heterogeneous gap and cellular heterogeneity is illustrated in Fig. 1, available as supplementary data at *Bioinformatics* online). (ii) Existing approaches rely on node feature and its neighbors to obtain the attention scores, which ignore the structural groupings (also known as graph clusters) for characterizing the topological structure hidden in scRNA-seq data.

In this paper, we propose a novel differentiable graph clustering with structural grouping (DGCSG), which incorporates graph cluster information into attention mechanism of graph attention autoencoder (GATE) by devising a differentiable graph clustering objective. Specifically, we first construct a cell–cell graph by applying Pearson correlation analysis and network-enhanced denoising (Wang *et al.* 2018) to the scRNA-seq data after quality control. Subsequently, to address the heterogeneous gap between cell node and their structural information, we build an interactive module based on AE and GATE, which dynamically fuses the cell node representations learned by AE into GATE in layer-by-layer manner. Thus, the structural features captured by GATE can be promoted by the node features of AE. Afterward, to incorporate graph cluster into deep graph clustering, we design a differentiable clustering mechanism to transform K-way normalized cuts from a discrete optimization problem into a differentiable learning objective through spectral relaxation. This differentiable clustering objective can be jointly optimized with GATE to allocate more attention scores to cell nodes in the same graph cluster, providing better cell representation for downstream clustering. Finally, a decoupled self-supervised optimization is proposed, which guides the representation learning of AE and GATE in the interactive module through two high-confidence distributions. Our contributions can be summarized as follows:

- To characterize graph cluster information of cell–cell graph, a novel differentiable clustering mechanism is proposed to transform K-way normalized cuts from a discrete optimization problem into a differentiable learning objective through spectral relaxation.
- A layer-by-layer interactive module based on AE and GATE is devised to deal with the heterogeneous gap between cell node and structural information, while a decoupled self-supervised optimization is designed to enhance the representation of scRNA-seq data by the module.
- Experimental evaluations on 14 scRNA-seq benchmarks validates that the proposed method significantly outperforms existing state-of-the-art baselines.

## 2 Materials and methods

Suppose we have access to unlabeled scRNA-seq data $X=\left\{x_{ij}\right\}\in R^{N\times M}$, where *N* and *M* are the number of cell and its gene expression count, $x_{ij}$ indicates the expression value of the *j*th gene in the *i*th cell. To capture the topological information of cells, we construct a cell–cell graph G=(X,A) with Pearson coefficient matrix, in which the degree matrix $D=\text{diag}(d_{1},d_{2},\ldots ,d_{N})$ can be calculated by $D_{ii}=\sum_{j}{\left(A\right)}_{ij}$, where A is the adjacency matrix. The proposed DGCSG aims to allocate scRNA-seq data into different subpopulations by characterizing cell node feature and its structural information derived by graph clusters simultaneously. The model architecture of DGCSG is shown in Fig. 1, which consists of the following components: data preprocessing, interactive module, differentiable graph clustering and decoupled self-supervised optimization. Specifically, in data preprocessing, we construct initialized cell–cell graph G=(X,A) with Pearson coefficient matrix between cells, then perform network enhancement to remove noisy edges of the graph. Note 1, available as supplementary data at *Bioinformatics* online, shows the construction process of the cell–cell graph. In differentiable graph clustering, to incorporate graph cluster information into deep graph clustering framework, the K-way normalized cuts problem is transformed from a discrete optimization problem into a differentiable learning objective $L_{SG}$ through spectral relaxation. Then, the interactive module is composed of AE and GATE with *L* layers, in which the node representation Z of AE is transferred into corresponding layer of GATE. By integrating differentiable graph clustering objective $L_{SG}$, GATE learns a representation H of cells that captures both node feature and graph cluster simultaneously. Finally, a decoupled self-supervised optimization is proposed, which guides the representation learning of AE and GATE in the interactive module through two high-confidence distributions.

### 2.1 Differentiable graph clustering with structural grouping

In graph clustering, graph cluster is an important structural information except for the neighbors of cell nodes, which can describe the structural relationships between cells more comprehensively. Thus, it is potential to introduce graph cluster information into self-attention mechanism, which can assign higher attention scores to cell nodes within the same cluster. In the literature, K-way normalized cuts (Shi 2003) method is an effective graph clustering approach, which is capable of dividing the original graph into multiple subgraphs. In this study, we resort K-way normalized cuts to partition cell nodes X into K disjoint graph clusters, i.e. $V=\cup_{i=1}^{K}C_{i}$ and $C_{i}\cap C_{j}=\emptyset ,\forall i\neq j$, by maximizing intracluster links and minimizing intercluster links as follows:

(1) $$\frac{1}{K}\sum_{k=1}^{K}\frac{\mathrm{links}\left(C_{k},C_{k}\right)}{\mathrm{degree}\left(C_{k}\right)}=\frac{1}{K}\sum_{k=1}^{K}\frac{\sum_{i,j\in C_{k}}A_{i,j}}{\sum_{i\in C_{k},j\in V}A_{i,j}},$$

where $C_{i}$ is the *i*th cell cluster, $\mathrm{links}\left(C_{k},C_{k}\right)$ is the number of links in cluster $C_{k}$, $\mathrm{degree}\left(C_{k}\right)$ is the number of links connected to $C_{k}$. To learn the graph cluster, we use $F\in {\left\{0,1\right\}}^{N\times K}$ to indicate the cluster assignment matrix, where $F_{i,j}=1$ means that the *i*th cell node is allocated into *j*th graph cluster, and $F_{i,j}=0$ means that it is not in this graph cluster. Now, the K-way normalized cuts problem [Equation (1)] is rewritten as follows:

(2) $$\begin{array}{c} \max\frac{1}{K}\sum_{k=1}^{K}\frac{F_{k}^{T}AF_{k}}{F_{k}^{T}DF_{k}},\\ \mathrm{subject}\;to\;F\in {\left\{0,1\right\}}^{N\times K},F1_{K}=1_{N}, \end{array}$$

where $F_{k}$ is the *k*th column of F, $F1_{K}=1_{N}$ indicates that each node can only belong to one cluster.

As shown in Equation (2), the cluster assignment matrix F of the K-way normalized cuts problem is a discrete variable, and the optimization of this variable is NP Hard (Shi and Malik 2000). Moreover, this discrete variable cannot be solved by gradient descent in deep graph clustering model. To solve this problem, we transform the discrete variable optimization problem of K-way normalized cuts into a continuously differentiable form using spectral relaxation method. From Equation (2), we can observe that the objective function of the K-way normalized cuts is a generalized Rayleigh quotient, which can be optimized by transforming it into a spectral decomposition problem of the matrix (Li 2015). Therefore, we first introduce a variable $E_{k}$ and set $E_{k}=D^{1/2}F_{k}{\left(F_{k}^{T}DF_{k}\right)}^{-1/2}$ to transform Equation (2) into the form of a standard Rayleigh quotient,

(3) $$\begin{array}{c} \max\frac{1}{K}\sum_{k=1}^{K}\frac{E_{k}^{T}D^{-1/2}AD^{-1/2}E_{k}}{E_{k}^{T}E_{k}},\\ \mathrm{subject}\;to\;E_{k}=D^{1/2}F_{k}{\left(F_{k}^{T}DF_{k}\right)}^{-1/2},E_{k}^{T}E_{k}=1, \end{array}$$

where $E_{k}$ is constrained by $E_{k}^{T}E_{k}=1$. Next, we relax the discreteness condition by allowing $E_{k}$ to take arbitrary real values and treat it as a soft clustering partition. To further simplify the problem, we provide the following proposition to transform the objective function of K-way normalized cuts into the problem of solving the K largest eigenvalues.

Proposition 1. *After relaxing the discreteness condition, the K-way normalized cuts problem can be transformed into the maximization of the average sum of the K eigenvalues by applying the Lagrange multiplier method.*

The proof of Proposition 1 is provided in Proof 1, available as supplementary data at *Bioinformatics* online. By utilizing the above proposition, the objective function of K-way normalized cuts can be written as follows:

(4) $$\max\frac{1}{K}\sum_{k=1}^{K}\lambda_{k},$$

where $\lambda_{k}$ is the eigenvalue of the matrix $D^{-1/2}AD^{-1/2}$ and the corresponding eigenvector is $E_{k}$. However, it is computationally expensive to directly solve the eigenvalues, especially for matrices with large dimensions. The Fan (1949) theorem states that maximizing the sum of eigenvalues can be achieved simply by finding the corresponding eigenvectors. We resort the Fan theorem to transform the problem into an optimization of a subset of eigenvectors, thereby avoiding the computation of the full eigenvalue spectrum.

Theorem 1 **(Fan).** *Let* $B\in R^{n\times n}$  *is a symmetric matrix, the eigenvalues of* B  *is*  $\lambda_{\text{max}}=\lambda_{1}\geq \lambda_{2}\geq \cdots \geq \lambda_{n-1}\geq \lambda_{n}=\lambda_{\text{min}}$  *with corresponding eigenvectors* $u_{1},u_{2},\ldots ,u_{n}$*, we can obtain*,

(5) $$\lambda_{1}+\cdots +\lambda_{k}=\max tr\left(W^{T}BW\right),$$

where $W^{*}=\left[u_{1},\ldots ,u_{k}\right]O$ is the optimal solution, and O is orthogonal.

 Finally, we apply the Fan theorem to convert Equation (4) into the following form:

(6) $$\begin{array}{c} \max\frac{1}{K}tr\left(E^{T}D^{-1/2}AD^{-1/2}E\right)\\ \mathrm{subject}\;to\;E=D^{1/2}F{\left(F^{T}DF\right)}^{-1/2},E^{T}E=I. \end{array}$$

The optimal solution of Equation (6) is the eigenvectors corresponding to the K largest eigenvalues of $D^{-1/2}AD^{-1/2}$. By relaxing the K-way normalized cuts to a Top-K eigenproblem (Sgherzi *et al.* 2021), we can transform discrete matrix F into a continuous matrix E, in which E can be treated as the soft partition matrix. Now, the continuous matrix can be parameterized using GATE layers.

### 2.2 Interactive module between cell node and graph structural information

GATE can assign different attention scores to different cell neighbors, which helps to learn cell representations. However, the existing GATE and its variants only consider information about the node and its neighbors to allocate the attention scores. In this study, we design a differentiable graph clustering mechanism to incorporate structural grouping information into the GATE attention learning process. However, the cell node feature is also an indispensable cue in scRNA-seq clustering for characterizing gene expression information. To simultaneously learn the node and structural information of cells, we devise an interactive module based on AE and GATE. Meanwhile, in order to eliminate the heterogeneous gap between cell nodes and their structural information, we dynamically fuse the learned representations in a layer-by-layer manner.

Firstly, we build an AE with *L* layers to capture the cell node features. The corresponding construction process can be found in Note 2, available as supplementary data at *Bioinformatics* online. Next, to combine cell node information and structural information driven by graph clusters, we construct a GATE that has the same number of layers as AE. Thus, the representation $h_{i}^{\left(l\right)}$ of cell node $x_{i}$ in the *l*th layer of GATE is formulated as follows:

(7) $$h_{i}^{\left(l\right)}=\sigma \left(\sum_{j\in N_{i}}att_{i,j}W_{\mathrm{gat}}h_{j}^{\left(l-1\right)}\right),$$

where $N_{i}$ is the neighbors of cell node $x_{i}$, σ(·) is ReLU function, $W_{\mathrm{gat}}$ is a learnable parameter. $att_{i,j}$ is the attention score between cell nodes $x_{i}$ and $x_{j}$, which can be calculated as follows:

(8) $$att_{i,j}=\frac{\;\text{exp}\;\left(\mathrm{LeakyReLU}\left(a^{T}\left(W_{\mathrm{gat}}h_{i}||W_{\mathrm{gat}}h_{j}\right)\right)\right)}{\sum_{k\in N_{i}}\;\text{exp}\;\left(\mathrm{LeakyReLU}\left(a^{T}\left(W_{\mathrm{gat}}h_{i}||W_{\mathrm{gat}}h_{j}\right)\right)\right)},$$

where *a* denotes a vector of weights. || represents the concatenation of two vectors. Thus, the node representation learned by AE is transferred layer by layer to corresponding layer in GATE for dynamic fusion in the following form:

(9) $$U^{\left(r\right)}=Z^{\left(r\right)}+H^{\left(r\right)},$$

where r∈{0,1,…,L} represents the number of encoder layers, and $Z^{\left(r\right)}$ and $H^{\left(r\right)}$ are the representations of the *r*th layer in AE and GATE, respectively, with $Z^{\left(0\right)}=H^{\left(0\right)}=X$. After dynamic fusion with the node representation learned by AE, the learned representation of the *l*th layer in GATE is expressed in the following form:

(10) $$H^{\left(l\right)}=\sigma \left(\mathrm{att}\cdot W_{\mathrm{gat}}^{\left(l\right)}U^{\left(l-1\right)}\right).$$

To incorporate the graph cluster information into GATE, we use $S=\mathrm{att}\cdot W_{\mathrm{gat}}^{\left(l\right)}U^{\left(l-1\right)}$ to parameterize the graph cluster information E in Equation (6). Then, the objective function of K-way normalized cuts can be incorporated into GATE as follows:

(11) $$\begin{array}{c} \max\frac{1}{K}tr\left(S^{T}D^{-1/2}AD^{-1/2}S\right),\\ \mathrm{subject}\;to\;S^{T}S=I. \end{array}$$

Now, we define the differentiable graph clustering loss for the *l*th layer and normalize it to obtain as follows:

(12) $$L_{SG}^{\left(l\right)}={\left\|\frac{1}{N}S^{T}S-I\right\|}_{F}^{2}-\frac{1}{NK}tr\left(S^{T}D^{-1/2}AD^{-1/2}S\right).$$

By combining the differentiable graph clustering loss of each layer in GATE, we can obtain the total differentiable graph clustering loss as follows:

(13) $$L_{SG}=\sum_{l=1}^{L}L_{SG}^{\left(l\right)}.$$

We reconstruct the cell–cell graph by designing an inner product decoder to decode the learned representations of GATE and define the reconstruction loss of GATE as follows:

(14) $$\begin{array}{c} \hat{A}=\mathrm{sigmoid}\left(H^{(L)T}H^{\left(L\right)}\right),\\ L_{\mathrm{GATEres}}=\mathrm{MSEloss}(A,\hat{A}). \end{array}$$

The reconstruction loss of the interactive module is calculated as the sum of the reconstruction losses of AE and GATE,

(15) $$L_{\mathrm{res}}=L_{\mathrm{AEres}}+L_{\mathrm{GATEres}},$$

where $L_{\mathrm{AEres}}$ is the reconstruction loss of AE and the specific definition can be found in Note 3, available as supplementary data at *Bioinformatics* online.

### 2.3 Decoupled self-supervised clustering loss

Self-supervised learning empowers the model to learn discriminative representations by mining deep semantic information from massive unlabeled data. In the self-supervised clustering literature, dual-channel networks often are optimized under the supervision of a common highly confidence signal derived from Student’s *t*-distribution on both the AE and GNN channels, such as Yan *et al.* (2023) and Bo *et al.* (2020). However, due to the uniqueness of features learned from different channels, a common high-confidence distribution cannot effectively guide the learning process of two channels. In this subsection, a decoupled self-supervised clustering loss is formulated to simultaneously guide the representation learning of AE and GATE in the interactive module.

For the learned representations $Z^{\left(L\right)}$ and $H^{\left(L\right)}$ of AE and GATE, we first utilize K-means to initialize the cluster centers *c* for the learned cell node representations $Z^{\left(L\right)}$. Then, the similarity between cell representations and cluster centers can be transformed into cluster assignments $Q^{z}$ and $Q^{h}$ based on Student’s *t*-distribution, as defined by the following formula:

(16) $$q_{ij}=\frac{{\left({\left\|z_{i}-c_{j}\right\|}^{2}+1\right)}^{-1}}{\sum_{k=1}^{K}{\left({\left\|z_{i}-c_{k}\right\|}^{2}+1\right)}^{-1}},$$

where $z_{i}$ represents the learned representation of cell $x_{i}$, and $c_{j}$ represents the *j*th cluster center.

Then, the enhanced cluster assignments $P^{z}=\left[p_{ij}^{z}\right]\in R^{N\times K}$ and $P^{h}=\left[p_{ij}^{h}\right]\in R^{N\times K}$ are generated by quadratically operating the soft cluster assignments $Q^{z}$ and $Q^{h}$, providing a more reliable supervisory signal for the training process (Wang *et al.* 2019). Specifically, the enhanced cluster assignments $P^{z}$ and $P^{h}$ are calculated as follows:

(17) $$p_{ij}^{z}=\frac{q_{ij}/\sum_{i=1}^{N}q_{ij}}{\sum_{k=1}^{K}\left(q_{ik}^{2}/\sum_{i=1}^{N}q_{ik}\right)},$$

where $P^{z}$ and $P^{h}$ can be seen as clustering assignments with higher confidence that $Q^{z}$ and $Q^{h}$ (the detailed explanation of why $P^{z}$ and $P^{h}$ can be treated as high-confidence assignments to guide clustering is presented in Note 4, available as supplementary data at *Bioinformatics* online). Thus, in each iteration, we minimize the Kullback–Leibler (KL) to make these two clustering assignments closer to each other, which ensures the clustering consistency between cluster assignment and its enhanced one according to the following equation:

(18) $$\begin{array}{c} L_{KL}^{z}=KL\left(P^{z}||Q^{z}\right)=\sum_{i}\sum_{j}p_{ij}^{z}\;\text{log}\;\left(p_{ij}^{z}/q_{ij}^{z}\right),\\ L_{KL}^{h}=KL\left(P^{h}||Q^{h}\right)=\sum_{i}\sum_{j}p_{ij}^{h}\;\text{log}\;\left(p_{ij}^{h}/q_{ij}^{h}\right). \end{array}$$

Now, the overall loss function of the proposed DGCSG consists of reconstruction loss [Equation (15)], decoupled self-supervised clustering loss [Equation (18)] and differentiable graph clustering loss [Equation (13)], which can be formulated as follows:

(19) $$L_{\mathrm{overall}}=L_{\mathrm{res}}+L_{KL}^{z}+\alpha L_{SG}+\beta L_{KL}^{h},$$

where α, β are the tradeoff parameters. This loss function combines data reconstruction, differentiable structural grouping information, and decoupled self-supervised optimization together, thus promoting the clustering performance.

### 2.4 Datasets and evaluation metrics

DGCSG is evaluated on 14 real scRNA-seq datasets, which include Klein (Klein *et al.* 2015), Chung (Chung *et al.* 2017), Sun.1, Sun.2, Sun.3 (Sun *et al.* 2019), Biase (Biase *et al.* 2014), Muraro (Muraro *et al.* 2016), Deng (Deng *et al.* 2014), Pollen (Pollen *et al.* 2014), Darmanis (Darmanis *et al.* 2015), 10X_PBMC (Zheng *et al.* 2017), Mouse bladder cells (Han *et al.* 2018), Habib (Habib *et al.* 2017), and Zeisel (Zeisel *et al.* 2015). Table 1, available as supplementary data at *Bioinformatics* online, provides detailed information about these datasets. The cells in these datasets vary from different organisms, organs and platforms, and their numbers range from tens to thousands.

In this study, we adopt two widely adopted clustering metrics including the Adjusted Rand Index (ARI) (Hubert and Arabie 1985) and the normalized mutual information (NMI) (Strehl and Ghosh 2002) to quantify the similarity between the predicted results and the real cell types. The higher the value of the metric, the higher the similarity. The definition and calculation process of clustering metrics are presented in Note 5, available as supplementary data at *Bioinformatics* online.

## 3 Results

### 3.1 Clustering performance analysis

To verify that DGCSG has excellent clustering performance, we compare the proposed method with 16 state-of-the-art clustering approaches. The comparison approaches include four graph clustering methods, i.e. CCGC (Yang *et al.* 2023), MBN (Yan *et al.* 2023), SDCN (Bo *et al.* 2020), SCGC (Kulatilleke *et al.* 2025), and 12 scRNA-seq clustering methods which include contrastive clustering methods, i.e. contrastive-sc (Ciortan and Defrance 2021), scNAME (Wan *et al.* 2022), nsDCC (Wang *et al.* 2024), deep embedded clustering methods, i.e. scDeepCluster (Tian *et al.* 2019), scziDesk (Chen *et al.* 2020), scSMD (Cui *et al.* 2025), deep graph clustering methods, i.e. scGNN (Wang *et al.* 2021), scGAC (Cheng and Ma 2022), scDFC (Hu *et al.* 2023), scTAG (Yu *et al.* 2022), scLEGA (Liu *et al.* 2024b), scDFN (Liu *et al.* 2024a). Among these approaches, scGAC and scDFC apply GATE for clustering. The scDFC, scDFN, and scLEGA use dual-channel networks integrating an AE and a GNN module to learn cell representations for clustering. The key implementation and configuration details of DGCSG and the comparison algorithms are presented in Note 6, available as supplementary data at *Bioinformatics* online. To ensure fairness, all approaches are repeatedly performed 10 times to report the average scores of ARI and NMI metrics.

**Figure 2.**
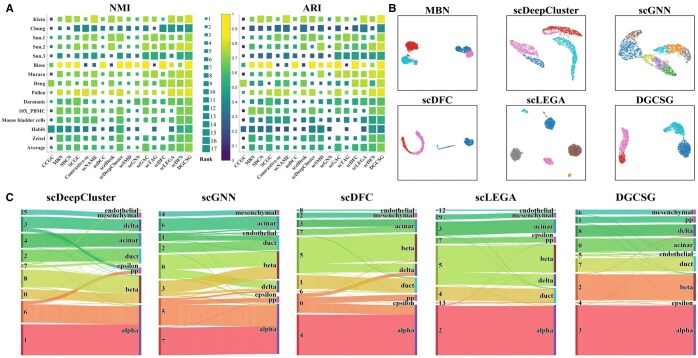
(A) NMI and ARI scores of DGCSG and 16 baselines on the 14 datasets. The performance of each baseline on each dataset is represented by a block, where the color and size of the block indicate the score and rank. The average performance of each baseline on all datasets is shown in the last row. In some places, there is no block because of out of memory. (B) Cell representations learned by DGCSG and five baselines on the Klein dataset. The color represents the predicted labels of each baseline. (C) The Sankey diagram shows the correspondence between the predicted results (on the left) and the real cell types (on the right) of DGCSG and four baselines on the Muraro dataset.

From Fig. 2A, it can be observed that DGCSG achieves the highest NMI scores on 12 datasets and the highest ARI scores on 13 datasets. Notably, for the ARI and NMI metrics, we calculate the average scores of each baseline on all datasets, and DGCSG achieves the highest scores on both metrics.

Tables 2 and 3, available as supplementary data at *Bioinformatics* online, present the detailed NMI and ARI scores for the comparison results, respectively. From the result, we get the follow observations: (i) DGCSG is superior to the deep embedded clustering baselines. For example, DGCSG obtains 17% and 22% improvements in terms of the ARI metric compared with scDeepCluster and scziDesk, respectively. This phenomenon shows that cell–cell structural relationship is an important cue for scRNA-seq clustering. (ii) Leveraging dual-channel network structure (MBN, SDCN, scDFN, scLEGA, and scDFC) to capture node and structural information of cells cannot consistently improve clustering performance. This is mainly because these baselines only fuse the learned cell node representations and structural representations, without addressing the heterogeneous gap between cell node feature and their structural information. In contrast, the interactive model in DGCSG dynamically integrates node and structural information in a layer-by-layer manner, achieving better clustering performance. (iii) Compared with GATE-based deep scRNA-seq clustering baselines (scDFC and scGAC), DGCSG achieves better clustering results. This is mainly because these baselines ignore the structural grouping information between cell nodes after constructing the cell–cell graph. In contrast, the differentiable graph clustering in our DGCSG assists GATE in allocating more attention scores to cell nodes in the same structural grouping, providing better cell representations for downstream clustering. In summary, DGCSG achieves superior clustering performance compared with other methods.

**Figure 3.**
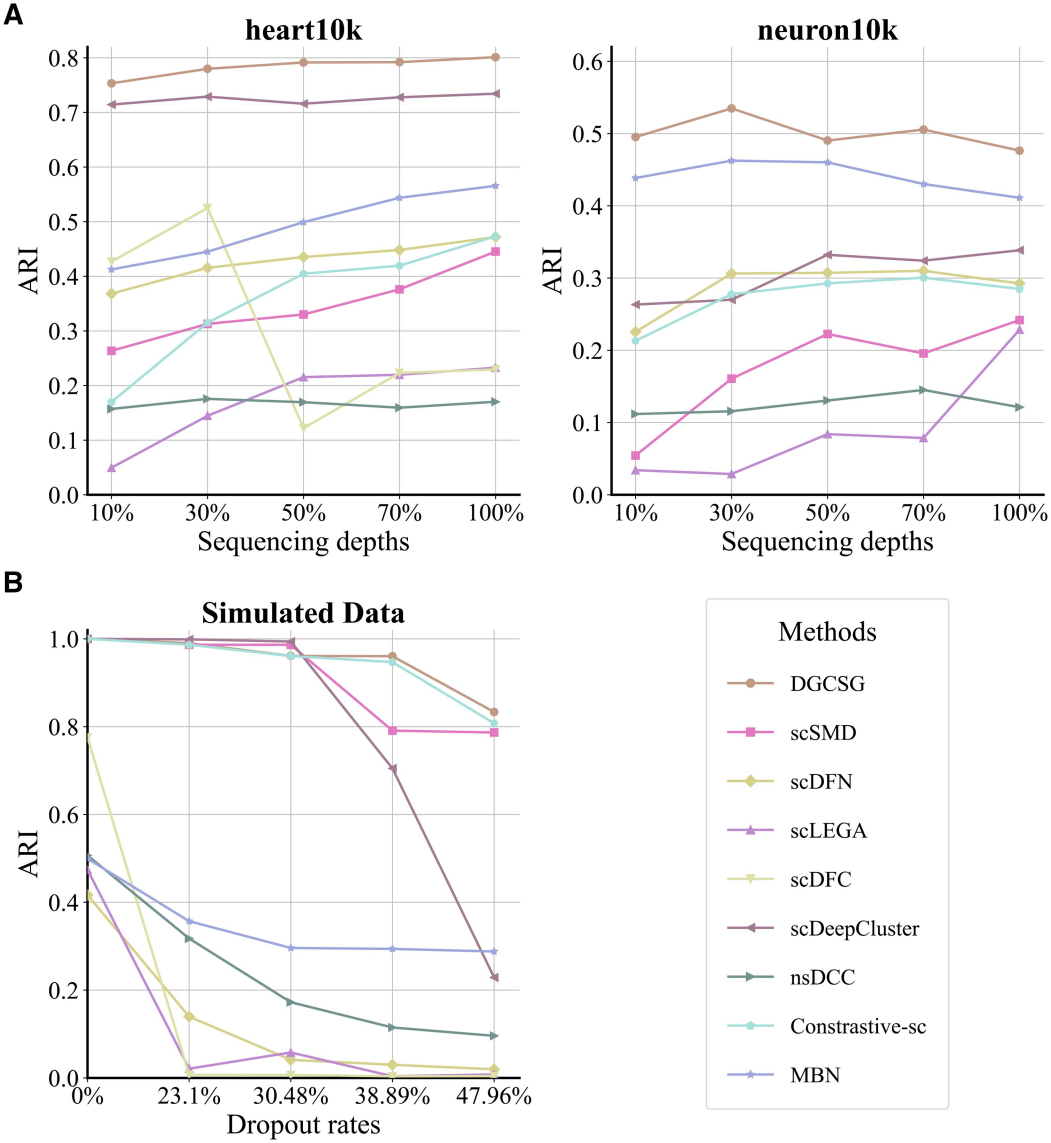
(A) ARI values of DGCSG and other baselines at different sequencing depths on heart10k dataset and neuron10k dataset. Note that scDFC is not included in the figure due to out-of-memory error. (B) ARI values of DGCSG and other baselines at different dropout rates on the simulated dataset.

Visualize the cell representations of the Klein dataset learned by DGCSG and five other baselines to intuitively understand the cell representations in low-dimensional space. As shown in Fig. 2B, the learned cell representations of DGCSG have clear boundaries and can separate different cell types well. In contrast, scDFC, scLEGA, scDeepCluster, and scGNN show limitations in clearly separating cells from different clusters, while MBN mixes cells from different clusters together. Visualizations of cell representations for some other datasets are shown in Figs 2 and 3, available as supplementary data at *Bioinformatics* online.

A Sankey diagram showing the correspondence between predicted results and real cell types is used to intuitively assess the accuracy of DGCSG and other four baselines, where the number of matches between predicted labels and true labels is represented by the width of the connecting lines. In this part, we select the Muraro dataset for visualization, which contains nine cell types: acinar, alpha, beta, epsilon, delta, duct, endothelial, mesenchymal, and pp. As shown in Fig. 2C, DGCSG generates nine predicted clusters, but the predicted cluster four fails to accurately correspond to epsilon cells. This is because there are only three epsilon cells in the Muraro dataset, and the small number makes accurate prediction more challenging. In contrast, scGNN and scLEGA incorrectly predict the number of clusters, while scDeepCluster and scDFC partition the same type of cells into different clusters.

### 3.2 Robustness analysis under various data sparsity levels

To evaluate the robustness of DGCSG on the single-cell data with various levels of sparsity, we perform experiments from the following two aspects: sequencing depth and dropout event.

**Figure 4.**
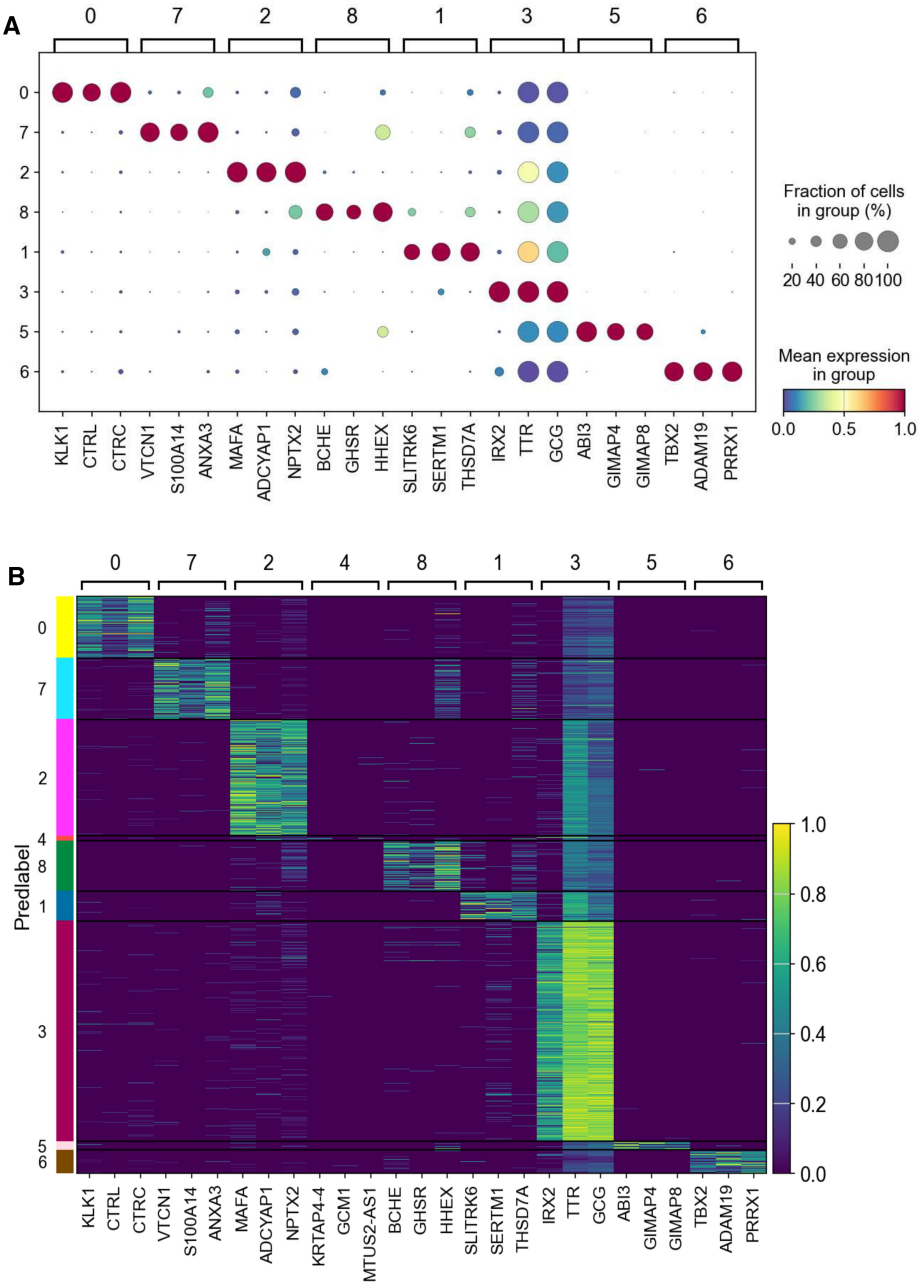
Expression levels of the top three DEGs identified by DGCSG for each predicted cluster. (A) Expression dot plot (Each dot represents the expression level of the gene in each predicted cluster. The color of each dot indicates the average expression value, while the size reflects the percentage of cells that are expressing the gene in each predicted cluster.). (B) Expression heat map.

To evaluate the robustness of DGCSG at different sequencing depths, we analyze its clustering performance by subsampling the sequencing depths of the heart10k and neuron10k datasets. The dataset descriptions and experimental implementation details are provided in Table 4 and Note 7, available as supplementary data at *Bioinformatics* online, respectively. The clustering performance of DGCSG with graph clustering, deep embedded clustering, contrastive clustering, attention mechanism based clustering, and dual-channel clustering baselines at different sequencing depths is shown in Fig. 3A and Fig. 4A, available as supplementary data at *Bioinformatics* online, and the corresponding NMI and ARI values are displayed in Tables 5–8, available as supplementary data at *Bioinformatics* online. From these figures, it can be observed that DGCSG outperforms other baselines in clustering performance under all sequencing depth conditions. DGCSG maintains superior performance even as the sequencing depth gradually decreases. In contrast, some baselines are sensitive to sequencing depth. For example, on the heart10k dataset, the clustering performance of scLEGA has a significant decrease of 18% in the ARI metric when the sequencing depth is reduced to 10% of the original depth. In summary, DGCSG shows better robustness under different sequencing depths.

In addition, we evaluate the impact of dropout events on the clustering performance of DGCSG by setting different levels of dropout rate in the simulated dataset. The descriptions of the dropout rate and simulated dataset, as well as the corresponding experimental settings are provided in Note 8, available as supplementary data at *Bioinformatics* online. The clustering performance of DGCSG with other baselines at different dropout rates is demonstrated in Fig. 3B and Fig. 4B, available as supplementary data at *Bioinformatics* online, and the corresponding NMI and ARI values are displayed in Tables 9 and 10, available as supplementary data at *Bioinformatics* online. From these figures, it can be observed that when the dropout rate is 38.89%, only the clustering metrics of DGCSG and contrastive-sc remain above 0.9. In contrast, the clustering performance of other methods such as scDFC and scLEGA is significantly degraded, with metrics close to 0. Furthermore, when the dropout rate reaches 47.96%, DGCSG still achieves optimal clustering performance, demonstrating its robustness in handling highly sparse scRNA-seq data, as well as its effectiveness in capturing the underlying cellular structure.

In summary, DGCSG achieves excellent clustering performance under different sparsity levels, verifying its robustness and superiority.

### 3.3 Ablation analysis

In this part, we conduct ablation experiments to verify the effectiveness of each module in DGCSG. There are five ablation variants: (i) removing the reconstruction loss, i.e. w/o $L_{\mathrm{res}}$, (ii) removing the AE module in the interactive module, i.e. w/o AE, (iii) removing the GATE module in the interactive module, i.e. w/o GATE, (iv) removing the differentiable graph clustering loss, i.e. w/o $L_{SG}$, (v) removing the decoupled self-supervised optimization module, and applying K-means clustering to the learned representations, i.e. w/o KL.

Figure 5A, available as supplementary data at *Bioinformatics* online, shows the ARI scores of these five variants with DGCSG on each dataset. The results indicate that for the majority of datasets, these variants result in either a decline or no significant change in clustering performance, with only a few datasets showing slight improvements. For example, removing the reconstruction loss slightly enhances the performance on the Sun.3 dataset, while removing GATE leads to marginal improvements on the Habib and 10X_PBMC datasets. Figure 5B, available as supplementary data at *Bioinformatics* online, shows the average ARI scores of DGCSG with five variants for 14 datasets, it can be observed that DGCSG outperforms these five variants in terms of clustering performance, which verifies the effectiveness of each module of DGCSG.

### 3.4 Model analysis

We explore the sensitivity of parameters α and β to the clustering performance of DGCSG. The value of α is taken from {3e-6,1e-5, 3e-5,1e-4, 3e-4}, while β takes values in the range {4, 16} with a step of 2, respectively. Figure 6, available as supplementary data at *Bioinformatics* online, shows the average results of 10 runs with different tradeoff parameters on all datasets. It can be seen that DGCSG achieves stable clustering on most of the datasets for different values of the tradeoff parameters. Based on these experimental results, we set α=3e-5, β=10 as the tradeoff parameters for all scRNA-seq datasets.

Besides, exploring model convergence is not only an important step to verify the correctness of the theory but also crucial for ensuring the dependability of the results and evaluating the stability of model training. As shown in Fig. 7, available as supplementary data at *Bioinformatics* online, we can observe that the objective function decreases and tends to converge with increasing of training iterations, while the clustering results increase and tend to converge. This phenomenon demonstrates that DGCSG has a good convergence property on single-cell scRNA-seq data.

In addition, we analyze the time complexity of DGCSG, which is detailed in Note 9, available as supplementary data at *Bioinformatics* online. The corresponding running time for DGCSG compared with 16 baselines on each dataset is presented in Table 11, available as supplementary data at *Bioinformatics* online. Since scGAC and scDFC are out of memory on the Habib dataset, we compute the average running times of DGCSG and the 16 baselines on the other 13 datasets. The result in Fig. 8, available as supplementary data at *Bioinformatics* online, demonstrates the superiority of DGCSG in terms of runtime.

### 3.5 Significance test

To verify that DGCSG achieves a significant improvement in clustering performance compared with existing baselines, we conduct a one-sided paired *t*-test of the experimental results on the ARI metrics and report the corresponding *P*-values. Specifically, for the experiments where DGCSG achieves the best performance, we define the null hypothesis as $H_{0}:ARI_{\mathrm{DGCSG}}\leq ARI_{\text{baseline}}$. For experiments where DGCSG does not achieve the optimal result, we define the null hypothesis as $H_{0}:ARI_{\text{baseline}}\leq ARI_{\mathrm{DGCSG}}$ to verify whether the baseline outperforms DGCSG. It is important to point out that DGCSG and several baselines obtained clustering performance with ARI = 1 on Biase dataset, thus no significance test is performed on this dataset. Table 12, available as supplementary data at *Bioinformatics* online, summarizes the significance test results of each method on different datasets, with bolded values representing the *P*-values where DGCSG fails to significantly outperform the baseline. Specifically, scDFN significantly outperforms DGCSG on the Habib dataset (*P*<.05), and DGCSG does not significantly outperform scSMD on the Muraro and Habib datasets (*P*>.05). However, on all the remaining datasets, DGCSG significantly outperforms the existing baselines. The above results validate the superiority of DGCSG in single-cell clustering tasks.

## 4 Biological analysis

### 4.1 Cell trajectory inference

Cell trajectory inference reconstructs the continuous trajectory of cells in the transcriptional space, providing a powerful tool for studying complex biological processes such as organ regeneration, cell differentiation, and embryonic development. In the literature, scCRT (Shi *et al.* 2024) is a novel and effective cell trajectory analysis method, which can perform cell trajectory inference based on the learned cell representations in low-dimensional space. In this experiment, we follow scCRT to perform cell trajectory inference on a synthetic dataset named binary_tree_8 with 8429 cells and 531 genes.

To evaluate the effectiveness of DGCSG, we adopt Hamming–Ipsen–Mikhailov (HIM), F1 branches and F1 milestones as metrics to measure the similarity between inferred trajectories and real trajectories. Specifically, HIM measures the similarity between predicted topology and real values, while F1 branches and F1 milestones measure the accuracy of cell ordering along trajectory branches and trajectory milestones, respectively. In addition, the degree of linear correlation between pseudotime and true time is quantified by calculating the Pearson correlation coefficient. In this experiment, the visualization of the cell trajectory inference results is provided in Fig. 9, available as supplementary data at *Bioinformatics* online, which shows the branching evolution and the corresponding position of the cell in the trajectory, with the left color representing the cell type and the right color representing the pseudotime. From the figure, it can be observed that the trajectories and pseudotimes inferred from the representations learned by DGCSG closely match the true values. Besides, the cells from different clusters are also distributed along the cell trajectories. In contrast, the trajectories and pseudotime inferred from the representations learned by other baselines exhibit inaccuracies. From this experiment, we can conclude that DGCSG is beneficial for cell trajectory inference.

### 4.2 Marker gene identification

Marker genes provide important information for evaluating cell states and annotating cell types, which helps to reveal the biological functions of cells. In this part, we first utilize the Wilcoxon Rank Sum test (Rosner *et al.* 2006) to identify the top 50 differentially expressed genes (DEGs) for each predicted cluster. Then, we select the top three DEGs as more representative marker genes. Figure 4 shows the expression levels of these marker genes in each predicted cluster of the Muraro dataset. It can be observed that the marker genes and their expression are different for each cell cluster, and the marker genes are expressed at significantly higher levels in the corresponding cell clusters compared with other cell clusters. In addition, we evaluate the reliability of the top three DEGs by referencing the marker genes recorded in the CellMarker (Zhang *et al.* 2019) database. It is observed that the vast majority of marker genes identified based on DGCSG predictions are highly consistent with the known cell type marker genes. For example, the DEGs (KLK1, CTRL, CTRC) in cluster 0 predicted by DGCSG are marker genes for acinar cells. Although some DEGs are not matched to CellMarker, we observe that they are highly expressed in the corresponding clusters, suggesting that these genes can potentially serve as novel marker genes for specific cell types.

## 5 Discussion

In this paper, we propose a novel DGCSG for scRNA-seq data. Specifically, we first construct an interactive module to fuse the node and structural information of cells. Then, a differentiable clustering mechanism is devised to jointly optimize GATE to learn the structural grouping information among cells, allocating more attention scores to nodes in the same graph cluster. Finally, a decoupled self-supervised optimization module is designed to guide the clustering process with specific high-confidence distributions. Experiments on 14 real scRNA-seq datasets show the superiority of DGCSG compared with state-of-the-art baselines. In future, it is interesting to explore the applicability of DGCSG on single-cell multiomics data.

## Supplementary Material

btaf347_Supplementary_Data

## Data Availability

The data underlying this article are available in https://github.com/Xiaoqiang-Yan/DGCSG.
