# Supplementary material for: Differentiable graph clustering with structural grouping for single-cell RNA-seq data
Source: Bioinformatics. 2025 Jun 13;41(7):btaf347. doi: 10.1093/bioinformatics/btaf347 (PMC12212642; doi:10.1093/bioinformatics/btaf347)
Supplement: btaf347_Supplementary_Data [file btaf347_supplementary_data.pdf]

**Supplementary Materials For**

# Differentiable Graph Clustering with Structural Grouping for Single-cell RNA-seq Data

## Supplementary Proof

**Supplementary Proof 1.** Proof procedure for transforming K-way normalized cuts into the problem of solving the K largest eigenvalues.

## Supplementary Notes

**Supplementary Note S1.** The construction of cell-cell graph.

**Supplementary Note S2.** The construction of an autoencoder with $L$ layers.

**Supplementary Note S3.** The definition of the reconstruction loss used in the autoencoder.

**Supplementary Note S4.** The detailed explanation of P as a high-confidence assignment for clustering guidance.

**Supplementary Note S5.** The definition and calculation process of clustering metrics.

**Supplementary Note S6.** The key implementation and configuration details of DGCSG and the comparison algorithms.

**Supplementary Note S7.** The implementation of sequencing depth subsampling for the heart10k and neuron10k datasets.

**Supplementary Note S8.** The description of the dropout rate and the detailed setup of the dropout experiment.

**Supplementary Note S9.** Complexity analysis.

## Supplementary Tables

**Supplementary Table S1.** Details of 14 scRNA-seq datasets.

**Supplementary Table S2.** NMI scores of DGCSG and 16 baselines on 14 scRNA-seq datasets.

**Supplementary Table S3.** ARI scores of DGCSG and 16 baselines on 14 scRNA-seq datasets.

**Supplementary Table S4.** Details of the heart10k dataset and neuron10k dataset.

**Supplementary Table S5.** NMI values of DGCSG and other baselines at different sequencing depths on heart10k dataset.

**Supplementary Table S6.** NMI values of DGCSG and other baselines at different sequencing depths on neuron10k dataset.

**Supplementary Table S7.** ARI values of DGCSG and other baselines at different sequencing depths on heart10k dataset.

**Supplementary Table S8.** ARI values of DGCSG and other baselines at different sequencing depths on neuron10k dataset.

**Supplementary Table S9.** NMI values of DGCSG and other baselines at different dropout rates on the simulated dataset.

**Supplementary Table S10.** ARI values of DGCSG and other baselines at different dropout rates on the simulated dataset.

**Supplementary Table S11.** Running time (seconds) of DGCSG and 16 baselines on 14 scRNA-seq datasets.

**Supplementary Table S12.** Significance test results of clustering performance between DGCSG and baselines in terms of ARI.

## Supplementary Figures

**Supplementary Fig. S1.** The difference between cellular heterogeneity and representation heterogeneity.

**Supplementary Fig. S2.** Visualization of cell representations for the Muraro and Sun.1 datasets.

**Supplementary Fig. S3.** Visualization of cell representations for the Sun.2 and Sun.3 datasets.

**Supplementary Fig. S4.** NMI values of DGCSG and other baselines at different conditions.

**Supplementary Fig. S5.** Ablation study for DGCSG.

**Supplementary Fig. S6.** Sensitivity analysis of $\alpha$ and $\beta$ for DGCSG on 14 datasets.

**Supplementary Fig. S7.** Convergence analysis of DGCSG on 9 datasets.

**Supplementary Fig. S8.** Average running time (seconds) of DGCSG and 16 baselines on 13 datasets except Habib.

**Supplementary Fig. S9.** Visualization of cell trajectory inference using representations learned by DGCSG and 4 baselines.

**Supplementary Proof 1.** Proof procedure for transforming K-way normalized cuts into the problem of solving the K largest eigenvalues.

Firstly, we propose a proposition: *After relaxing the discreteness condition, the K-way normalized cuts problem can be transformed into the maximization of the average sum of the K eigenvalues by applying the Lagrange multiplier method.*

Next, we provide a proof for the proposition, where the K-way normalized cuts problem is given in the following form,

$$\begin{aligned} &\max \frac{1}{K}\sum_{k=1}^{K} \frac{E_k^T \mathbf{D}^{-1/2}\mathbf{A}\mathbf{D}^{-1/2}E_k}{E_k^T E_k}, \\ &\textit{subject to}\, E_k = \mathbf{D}^{1/2}F_k (F_k^T \mathbf{D} F_k)^{-1/2}, E_k^T E_k = 1. \end{aligned} \tag{1}$$

And for the following Equation,

$$\begin{aligned} &\max \frac{E_k^T \mathbf{D}^{-1/2}\mathbf{A}\mathbf{D}^{-1/2}E_k}{E_k^T E_k}, \\ &\textit{subject to}\, E_k = \mathbf{D}^{1/2}F_k (F_k^T \mathbf{D} F_k)^{-1/2}, E_k^T E_k = 1. \end{aligned} \tag{2}$$

Let $\mathbf{E} = [E_1, E_2, \cdots, E_K]$. It can be observed that Equation 1 is the summing and averaging operation of Equation 2 for the K column vectors of the matrix $\mathbf{E}$. Equation 2 is in the form of a Rayleigh quotient and has the constraint $E_k^T E_k = 1$. Therefore, we apply the Lagrange multiplier method to solve Equation 2 and construct the following objective function,

$$\mathcal{L}(E_k, \lambda_k) = \max_{E_k, \lambda_k}(E_k^T \mathbf{D}^{-1/2}\mathbf{A}\mathbf{D}^{-1/2}E_k - \lambda_k (E_k^T E_k - 1)). \tag{3}$$

By differentiating Equation 3 with respect to $E_k$ and $\lambda_k$, and set $\partial\mathcal{L}/\partial E_k = 0$, $\partial\mathcal{L}/\partial\lambda_k = 0$, we obtain the following eigenvalue decomposition form,

$$\mathbf{D}^{-1/2}\mathbf{A}\mathbf{D}^{-1/2}E_k = \lambda_k E_k, \tag{4}$$

where $\lambda_k$ is the eigenvalue of $\mathbf{D}^{-\frac{1}{2}}\mathbf{A}\mathbf{D}^{-\frac{1}{2}}$ and $E_k$ is the corresponding eigenvector. Substituting the above result into Equation 1, we obtain the following form,

$$\max \frac{1}{K}\sum_{k=1}^{K} \lambda_k. \tag{5}$$

This indicates that the objective of the optimization problem is to maximize the average of the top K eigenvalues.

**Supplementary Note S1.** The construction of cell-cell graph.

First, we perform quality control on the raw scRNA-seq data by filtering out low-quality cells with gene expression counts above the upper limit [75% of all cells plus 3 times the interquartile range] and below the lower limit [25% minus 3 times the interquartile range], as well as low-quality genes expressed in fewer than 3 cells. After quality control, the read counts (expression values) are normalized by dividing by the library size (total read counts of a cell), multiplying by 100,000, and log transforming with a pseudo count as 1.

Then, we set the PCA dimension to 2000, while for datasets with fewer than 2000 genes (Sun.1, Sun.2, Sun.3), the dimension is set to 512. If the number of cells is fewer than the PCA dimension, we select the top 2000 highly variable genes. After performing PCA, we obtain the gene expression matrix $\mathbf{X}$.

Finally, the Pearson coefficient matrix between cells is constructed based on the normalized scRNA-seq data, and network enhancement (NE) is performed on the matrix to remove noisy edges. Subsequently, for each cell, the $u$ most similar cells in the denoised matrix are selected and connected to construct the adjacency matrix. $u$ is calculated as follows,

$$u = \text{round}(\frac{N}{10 \times K}) \tag{6}$$

where $N$ is the number of cells, K is the number of cell clusters, and $u$ is specified to take values between 6 and 20.

**Supplementary Note S2.** The construction of an autoencoder with $L$ layers.

We build an autoencoder (AE) with $L$ layers to capture the cell node features. The representation of encoder and decoder of AE can be formulated as follows,

$$\mathbf{Z}^{(l)} = \sigma(\mathbf{W}^{(l)}\mathbf{Z}^{(l-1)} + \mathbf{b}^{(l)}), \tag{7}$$

$$\mathbf{Z}^{(l)} = \sigma(\mathbf{W}^{(l)}\mathbf{Z}^{(l-1)} + \hat{\mathbf{b}}^{(l)}), \tag{8}$$

where $l \in \{1, 2, \cdots, L\}$, $\sigma(\cdot)$ is ReLU function. $\mathbf{W}^{(l)}$, $\mathbf{Z}^{(l)}$, $\mathbf{b}^{(l)}$ and $\mathbf{W}^{l}$, $\mathbf{Z}^{(l)}$, $\hat{\mathbf{b}}^{(l)}$ are weight matrices, feature representations and biases in the encoder and decoder, respectively. We take the gene expression matrix $\mathbf{X}$ as the input for representation learning, i.e., $\mathbf{X} = \mathbf{Z}^{(0)}$. After reconstruction through the $L$ layers decoder, the reconstructed gene expression matrix is obtained, i.e., $\mathbf{X} = \mathbf{Z}^{(L)}$.

**Supplementary Note S3.** The definition of the reconstruction loss used in the autoencoder.

After obtaining the reconstructed gene expression matrix, we define the reconstruction loss of AE as follows,

$$\mathcal{L}_{AEres} = \frac{1}{2N} \| \mathbf{X} - \mathbf{X} \|_2^2 = \frac{1}{2N} \sum_{i=1}^{N} \| x_i - \hat{x}_i \|_2^2 \tag{9}$$

where $x_i \in \mathbf{X}$ and $\hat{x}_i \in \mathbf{X}$ indicate the original and reconstructed cell data, respectively.

**Supplementary Note S4.** The detailed explanation of P as a high-confidence assignment for clustering guidance.

In this study, a decoupled self-supervised module is designed for guiding the learning of representations through high-confidence assignments during training. Specifically, we apply student's t-distribution to calculate the soft cluster assignments $\mathbf{Q}^z$ and $\mathbf{Q}^h$, and construct the enhanced cluster assignments $\mathbf{P}^z$ and $\mathbf{P}^h$ based on $\mathbf{Q}^z$ and $\mathbf{Q}^h$. By squaring $\mathbf{Q}^z$ and $\mathbf{Q}^h$ when constructing $\mathbf{P}^z$ and $\mathbf{P}^h$, we can enhance the weight of high-confidence samples and suppress low-confidence samples, thus providing a more reliable supervisory signal for the training process. This mechanism enables the model to give priority to more credible clustering structures during the optimization process and improve the clustering quality.

In addition, the normalization of the soft assignments of each cluster is introduced when constructing $\mathbf{P}^z$ and $\mathbf{P}^h$, which effectively balances the contribution of different clusters in the loss function. At the same time, it also avoids the problem that some clusters dominate the training process due to a large number of samples, and improves the stability and robustness of the model under complex clustering structures.

**Supplementary Note S5.** The definition and calculation process of clustering metrics.

Normalized mutual information (NMI) is a metric used to evaluate the degree of association between clustering results and true labels. It is based on mutual information (MI) in information theory, and facilitates the comparison between different clustering results by standardizing the MI to keep its value in the [0,1] interval. The NMI is calculated as follows,

$$\mathrm{NMI} = \frac{2MI(U,V)}{H(U)+H(V)}, \tag{10}$$

where $U$ represents the clustering results, $V$ denotes the truth labels, $MI(U,V)$ indicates the mutual information between $U$ and $V$, and $H$ is the entropy function. The larger value of NMI indicates that the clustering result is closer to the true label.

Adjusted rand index (ARI) is a statistical metric used to measure the similarity between clustering results and true labels. Different from the original Rand Index (RI), ARI corrects for the expected similarity of random divisions, thus eliminating the similarity bias due to random clustering. Its formula is as follows,

$$\mathrm{ARI} = \frac{\sum_i \sum_j \binom{n_{ij}}{2} - \left[\sum_i \binom{a_i}{2} \sum_j \binom{b_j}{2}\right] / \binom{n}{2}}{\frac{1}{2}\left[\sum_i \binom{a_i}{2} + \sum_j \binom{b_j}{2}\right] - \left[\sum_i \binom{a_i}{2} \sum_j \binom{b_j}{2}\right] / \binom{n}{2}}, \tag{11}$$

where $n$ represents the total number of samples, $n_{ij}$ represents the number of samples in the intersection of the $i$-th true label and the $j$-th clustering result. $a_i$ denotes the number of samples in the $i$-th truth label, and $b_j$ denotes the number of samples in the $j$-th clustering result. The ARI value ranges from [−1,1], with a higher value indicating better clustering performance.

**Supplementary Note S6.** The key implementation and configuration details of DGCSG and the comparison algorithms.

We implement DGCSG using PyTorch 1.12 and Python 3.9. DGCSG learns the node representations of cells through an autoencoder (AE), while simultaneously capturing the structural information of

cells via a graph attention encoder (GATE). The autoencoder consists of three linear transformation layers, with the output dimensions of the encoder being 500, 2000, and 10, respectively, and the decoder structure being symmetrical. To initialize the cluster centers, we perform 20 K-means clustering based on the latent representation learned by the pre-trained autoencoder, and select the best performing clustering result for initialization. Subsequently, we train the model for 400 epochs on 14 real scRNA-seq datasets with a learning rate of 1e-4. The parameters in the loss function are set as $\alpha$ = 10 and $\beta$ = 3e-5. All experiments are conducted on an RTX 4090 GPU (64 GB).

For the 4 graph clustering methods (i.e., CCGC, MBN, SDCN, SCGC), we adopt the same data preprocessing method as that of DGCSG. Specifically, we first filter out low-expressed genes and low-quality cells in the raw data. Then, we apply principal component analysis (PCA) to reduce the dimensionality of the gene expression matrix. Based on the reduced data, we compute the Pearson correlation matrix and employ the NE to remove noisy edges. Finally, we construct the cell-cell graph using the KNN algorithm (the detailed process is in Supplementary Note S1). For those methods designed for clustering scRNA-seq data, the raw data are directly used as input for preprocessing and training. The parameters for all methods are configured with their default values or according to their respective guidelines. We run each method 10 times and report the average results.

**Supplementary Note S7.** The implementation of sequencing depth subsampling for the heart10k and neuron10k datasets.

Specifically, we downloaded the FASTQ files of the heart10k and neuron10k datasets produced with the 10x Genomics Chromium platform (V3). The details of these datasets are provided in Supplementary Table S4. We then performed subsampling at 10%, 30%, 50%, 70%, and 100% of the original sequencing depth. The subsampling process was performed using the seqtk v1.3. For each subsample, we applied kallisto and bustools to construct the corresponding Unique Molecular Identifier (UMI) count matrix, where the mouse transcriptome and annotations were referenced from Ensembl release 96. The generated count matrix was used for the model training after the preprocessing process. The parameters $\alpha$ is set to 10 and $\beta$ is set to 3e-5, which were consistent with the setting of DGCSG on the other 14 scRNA-seq datasets.

**Supplementary Note S8.** The description of the dropout rate and the detailed setup of the dropout experiment.

We considered the expression matrix before dropout as the true count matrix and the matrix after dropout as the raw observed data. The dropout rate is defined as the proportion of false-zero counts to non-zero counts in the true count matrix due to low sequencing depth or other technical limitations in transcriptome sequencing. A higher dropout rate indicates higher sparsity in the data. The dropout rate is calculated as follows,

$$\text{dropout rate} = \frac{\text{Number of zeros in raw counts} - \text{Number of zeros in true counts}}{\text{Number of non-zero counts in true counts}} \tag{12}$$

In this section, we used the Splatter R package to generate simulated scRNA-seq datasets. Specifically, the splatSimulate function was applied to simulate the gene expression matrix of scRNA-seq data. To control the sparsity of the data, we set the dropout.mid parameter to range from 0 to 2 (i.e., 0, 0.5, 1, 1.5, 2), which corresponds to dropout rates of 0%, 23.1%, 30.48%, 38.89%, and

47.96%, respectively. We fixed the simulated data to 5 clusters, 2000 cells and 2500 genes. Following the setting of scDeepCluster, we set dropout.shape = -1 and de.fracScale = 0.3. The generated data were used as input to the models after preprocessing. The DGCSG and other baselines were run 10 times, and the average results were used for evaluation.

**Supplementary Note S9.** Complexity analysis.

The time complexity of the DGCSG mainly comes from the AE module, GATE module, differentiable graph clustering module and decoupled self-supervised optimization module. The time complexity of the AE module can be seen as $\mathcal{O}(N(\sum_{l=1}^{L} d_{l-1}d_l))$, where $N$ denotes the number of cells, $d_0$ denotes the dimension of the input data, and $d_l$ represents the dimension of the encoder at the $l$-th layer. For the GATE module, the time complexity is $\mathcal{O}(N(\sum_{l=1}^{L} d_{l-1}d_l)+|\varepsilon|(\sum_{l=1}^{L} d_l))$, where $|\varepsilon|$ represents the number of edges in the graph. The time complexity of the differentiable graph clustering module is $\mathcal{O}(N(\sum_{l=1}^{L} d_l^2)+|\varepsilon|(\sum_{l=1}^{L} d_l))$. For the decoupled self-supervised optimization module, its complexity is approximately $\mathcal{O}(NK+N\log N)$, where $K$ is the cluster number. In summary, the time complexity of the DGCSG is $\mathcal{O}(N(\sum_{l=1}^{L} d_{l-1}d_l+d_l^2)+|\varepsilon|(\sum_{l=1}^{L} d_l)+NK+N\log N)$.

**Supplementary Table S1.** Details of 14 scRNA-seq datasets.

| Datasets | Cells | Genes | Cell types | Platform | Reference |
|---|---|---|---|---|---|
| Klein | 2717 | 24175 | 4 | inDrop | [1] |
| Chung | 515 | 57915 | 5 | Smart-Seq | [2] |
| Sun.1 | 1756 | 1000 | 6 | Chromium | [3] |
| Sun.2 | 4853 | 1000 | 8 | Chromium | [3] |
| Sun.3 | 8218 | 1000 | 7 | Chromium | [3] |
| Biase | 49 | 25737 | 3 | Smart-Seq | [4] |
| Muraro | 2122 | 19046 | 9 | CEL-Seq2 | [5] |
| Deng | 268 | 22431 | 6 | Smart-seq | [6] |
| Pollen | 301 | 23730 | 11 | Smart-seq | [7] |
| Darmanis | 466 | 22088 | 9 | Smart-seq | [8] |
| 10X_PBMC | 4270 | 16652 | 8 | 10X Genomics | [9] |
| Mouse bladder cells | 2745 | 20669 | 16 | Microwell-Seq | [10] |
| Habib | 11148 | 23589 | 10 | Drop-Seq | [11] |
| Zeisel | 3004 | 19971 | 9 | STRT-Seq | [12] |

**Supplementary Table S2.** NMI scores of DGCSG and 16 baselines on 14 scRNA-seq datasets.

| Datasets | CCGC | MBN | SDCN | SCGC | Contrastive-sc | scNAME | nsDCC | scziDesk | scDC | scSMD | scGNN | scGAC | scTAG | scDFC | scLEGA | scDFN | DGCSG |
|---|---|---|---|---|---|---|---|---|---|---|---|---|---|---|---|---|---|
| Klein | 0.1524 | 0.7806 | 0.8540 | 0.8486 | 0.7545 | 0.9096 | 0.7719 | 0.8542 | 0.8448 | 0.7719 | 0.6838 | 0.8474 | 0.8310 | 0.6484 | 0.8780 | 0.8509 | **0.9351** |
| Chung | 0.2484 | 0.3960 | 0.3320 | 0.4524 | 0.4614 | 0.4523 | 0.4334 | 0.4296 | 0.4369 | 0.4334 | 0.4274 | 0.4477 | 0.2930 | 0.4909 | 0.5240 | 0.4528 | **0.5286** |
| Sun.1 | 0.5160 | 0.6687 | 0.6730 | 0.7251 | 0.8066 | 0.8068 | 0.6119 | 0.8071 | 0.8247 | 0.6119 | 0.6665 | 0.7868 | 0.8236 | 0.7960 | 0.7660 | 0.7574 | **0.8369** |
| Sun.2 | 0.3441 | 0.7294 | 0.7175 | 0.8085 | 0.7989 | 0.7596 | 0.6633 | 0.8425 | 0.6896 | 0.6633 | 0.6840 | 0.8573 | 0.7981 | 0.8272 | 0.8038 | 0.9016 | **0.9103** |
| Sun.3 | 0.3237 | 0.5086 | 0.4338 | 0.5081 | 0.5196 | 0.5487 | 0.4952 | 0.4008 | 0.5285 | 0.4952 | 0.4340 | 0.5891 | 0.6103 | 0.5880 | 0.4920 | 0.6013 | **0.6267** |
| Biase | 0.7328 | 1.0000 | 1.0000 | 1.0000 | 0.2059 | 0.3739 | 1.0000 | 0.1584 | 1.0000 | 1.0000 | 0.9702 | 1.0000 | 0.1061 | 1.0000 | 0.9290 | 0.8103 | **1.0000** |
| Muraro | 0.1770 | 0.7901 | 0.7435 | 0.7893 | 0.7730 | 0.8286 | 0.6837 | 0.7813 | 0.7549 | 0.6837 | 0.6351 | 0.8155 | 0.8417 | 0.7481 | 0.8330 | **0.8868** | 0.8786 |
| Deng | 0.7985 | 0.6868 | 0.7869 | 0.6174 | 0.7523 | 0.8023 | 0.6155 | 0.7918 | 0.6654 | 0.6155 | 0.7498 | 0.7916 | 0.6887 | 0.8250 | 0.8590 | 0.8116 | **0.8911** |
| Pollen | 0.7106 | 0.9172 | 0.8796 | 0.9239 | 0.9325 | 0.9078 | 0.8274 | 0.9052 | 0.9263 | 0.8274 | 0.8780 | 0.9034 | 0.8921 | 0.8697 | 0.9340 | 0.9248 | **0.9490** |
| Darmanis | 0.5406 | 0.6620 | 0.6822 | 0.7589 | 0.6403 | 0.6160 | 0.5523 | 0.5419 | 0.6273 | 0.5523 | 0.6896 | 0.7317 | 0.6253 | 0.6484 | 0.7420 | 0.7019 | **0.7877** |
| 10X_PBMC | 0.2270 | 0.7666 | 0.7395 | 0.7641 | 0.7368 | 0.7323 | 0.6391 | 0.7221 | 0.7740 | 0.6391 | 0.5108 | 0.7593 | 0.7344 | 0.6899 | 0.6160 | 0.7762 | **0.7829** |
| Mouse bladder | 0.2720 | 0.6975 | 0.6791 | 0.6933 | 0.7019 | 0.7145 | 0.5305 | 0.6215 | 0.7190 | 0.5305 | 0.6017 | 0.6696 | 0.6921 | 0.6083 | 0.6900 | **0.7504** | 0.7324 |
| Habib | 0.4957 | 0.4867 | 0.4626 | 0.4454 | 0.4124 | 0.3658 | 0.3172 | 0.3694 | 0.6380 | 0.3172 | 0.3553 | — | 0.3679 | — | 0.4035 | 0.6016 | **0.6552** |
| Zeisel | 0.1349 | 0.7182 | 0.6472 | 0.6854 | 0.5725 | 0.6153 | 0.4979 | 0.6041 | 0.7252 | 0.4979 | 0.5387 | 0.6337 | 0.5400 | 0.5167 | 0.6660 | 0.7367 | **0.7617** |
| Average | 0.4053 | 0.7006 | 0.6879 | 0.7157 | 0.6478 | 0.6738 | 0.6171 | 0.6307 | 0.7253 | 0.6170 | 0.6063 | 0.7357 | 0.6317 | 0.6945 | 0.7240 | 0.7546 | **0.8054** |

'—' indicates missing values, due to out-of-memory running of scGAC and scDFC on the Habib dataset. To calculate the average NMI scores of scGAC and scDFC across all datasets, missing values in the table are replaced by the mean NMI scores across other methods.

**Supplementary Table S3.** ARI values of DGCSG and 16 baselines on 14 scRNA-seq datasets.

| Datasets | CCGC | MBN | SDCN | SCGC | Contrastive-sc | scNAME | nsDCC | scziDesk | scDC | scSMD | scGNN | scGAC | scTAG | scDFC | scLEGA | scDFN | DGCSG |
|---|---|---|---|---|---|---|---|---|---|---|---|---|---|---|---|---|---|
| Klein | 0.0442 | 0.7808 | 0.8757 | 0.8389 | 0.7407 | 0.9027 | 0.7745 | 0.8552 | 0.8424 | 0.7860 | 0.5230 | 0.8269 | 0.7789 | 0.4705 | 0.8960 | 0.8380 | **0.9613** |
| Chung | 0.0934 | 0.2998 | 0.2975 | 0.2871 | 0.3457 | 0.3666 | 0.2566 | 0.3863 | 0.2859 | 0.3293 | 0.1838 | 0.3091 | 0.0990 | 0.3198 | 0.4190 | 0.2571 | **0.4735** |
| Sun.1 | 0.2781 | 0.5823 | 0.5952 | 0.6598 | 0.7819 | 0.7497 | 0.4865 | 0.7650 | 0.7849 | 0.7470 | 0.4441 | 0.7546 | 0.8274 | 0.7663 | 0.6900 | 0.6510 | **0.8563** |
| Sun.2 | 0.0772 | 0.6877 | 0.5433 | 0.6752 | 0.6821 | 0.5942 | 0.5784 | 0.7735 | 0.5163 | 0.8458 | 0.4961 | 0.8121 | 0.6782 | 0.6956 | 0.6900 | 0.8595 | **0.9215** |
| Sun.3 | 0.1332 | 0.3296 | 0.3509 | 0.3576 | 0.3212 | 0.4098 | 0.3493 | 0.2667 | 0.3351 | 0.3564 | 0.2330 | 0.5503 | 0.5051 | 0.5539 | 0.5030 | 0.5496 | **0.5728** |
| Biase | 0.6097 | 1.0000 | 1.0000 | 1.0000 | 0.1197 | 0.3615 | 1.0000 | 0.1693 | 1.0000 | 0.9482 | 0.9850 | 1.0000 | 0.0560 | 1.0000 | 0.9480 | 0.8294 | **1.0000** |
| Muraro | 0.0597 | 0.6682 | 0.6739 | 0.8004 | 0.6767 | 0.8094 | 0.6158 | 0.7136 | 0.6018 | 0.8622 | 0.4892 | 0.8715 | 0.8916 | 0.8440 | 0.8320 | 0.9234 | **0.9259** |
| Deng | 0.8497 | 0.5215 | 0.5614 | 0.2688 | 0.6442 | 0.6933 | 0.4666 | 0.7618 | 0.4113 | 0.3402 | 0.4768 | 0.5565 | 0.4959 | 0.8680 | 0.8770 | 0.6111 | **0.8917** |
| Pollen | 0.5210 | 0.8330 | 0.8227 | 0.8636 | 0.8841 | 0.8716 | 0.7476 | 0.8626 | 0.8753 | 0.7124 | 0.8177 | 0.8779 | 0.7925 | 0.8602 | 0.8510 | 0.9207 | **0.9509** |
| Darmanis | 0.3706 | 0.4777 | 0.5378 | 0.6902 | 0.5551 | 0.5217 | 0.3983 | 0.3782 | 0.4550 | 0.4921 | 0.5329 | 0.6591 | 0.4711 | 0.5762 | 0.6740 | 0.6453 | **0.7072** |
| 10X_PBMC | 0.0160 | 0.7944 | 0.7557 | 0.7436 | 0.6589 | 0.6717 | 0.5140 | 0.6195 | 0.7575 | 0.6393 | 0.3463 | 0.7118 | 0.6741 | 0.5433 | 0.4193 | 0.7578 | **0.7967** |
| Mouse bladder | 0.0197 | 0.4657 | 0.3867 | 0.5383 | 0.5330 | 0.5427 | 0.3159 | 0.4030 | 0.4942 | 0.3580 | 0.3893 | 0.3794 | 0.4735 | 0.3594 | 0.4980 | 0.5490 | **0.6130** |
| Habib | 0.3262 | 0.3832 | 0.3601 | 0.3843 | 0.2408 | 0.2595 | 0.1946 | 0.2301 | 0.4250 | 0.4335 | 0.2319 | — | 0.2276 | — | 0.3080 | **0.5480** | 0.4393 |
| Zeisel | 0.0346 | 0.6376 | 0.5222 | 0.6239 | 0.5351 | 0.5825 | 0.3582 | 0.5966 | 0.6204 | 0.6030 | 0.3423 | 0.5800 | 0.4938 | 0.4436 | 0.5850 | 0.7214 | **0.7307** |
| Average | 0.2452 | 0.6043 | 0.5916 | 0.6236 | 0.5513 | 0.5954 | 0.5040 | 0.5558 | 0.6003 | 0.6038 | 0.4636 | 0.6589 | 0.5331 | 0.6168 | 0.6564 | 0.6900 | **0.7743** |

'—' indicates missing values, due to out-of-memory running of scGAC and scDFC on the Habib dataset. To calculate the average ARI scores of scGAC and scDFC across all datasets, missing values in the table are replaced by the mean ARI scores across other methods.

**Supplementary Table S4.** Details of the heart10k dataset and neuron10k dataset.

| Datasets | Cells | Genes | Cell types | Total reads | Reads per cell | Download link |
|---|---|---|---|---|---|---|
| heart10k | 7713 | 36047 | 13 | 290439571 | 37655 | https://support.10xgenomics.com/single-cell-gene-expression/datasets/3.0.0/heart_10k_v3 |
| neuron10k | 11843 | 36047 | 16 | 357111595 | 30153 | https://support.10xgenomics.com/single-cell-gene-expression/datasets/3.0.0/neuron_10k_v3 |

**Supplementary Table S5.** NMI values of DGCSG and other baselines at different sequencing depths on heart10k dataset.

| Sequencing depths | MBN | Contrastive-sc | nsDCC | scDC | scSMD | scDFC | scLEGA | scDFN | DGCSG |
|---|---|---|---|---|---|---|---|---|---|
| 29043957 reads (10%) | 0.5798 | 0.4153 | 0.3706 | <u>0.7852</u> | 0.5083 | 0.6631 | 0.2970 | 0.5746 | **0.7981** |
| 87131871 reads (30%) | 0.5888 | 0.5479 | 0.3896 | <u>0.7929</u> | 0.5758 | 0.6951 | 0.4254 | 0.6458 | **0.8213** |
| 145219786 reads (50%) | 0.6110 | 0.5974 | 0.4051 | <u>0.7978</u> | 0.6121 | 0.4534 | 0.5720 | 0.6671 | **0.8257** |
| 203307700 reads (70%) | 0.6455 | 0.5995 | 0.4008 | <u>0.7932</u> | 0.6436 | 0.5771 | 0.5673 | 0.6734 | **0.8277** |
| 290439571 reads (100%) | 0.6642 | 0.6397 | 0.4104 | <u>0.7951</u> | 0.6550 | 0.6089 | 0.5436 | 0.6920 | **0.8404** |

**Supplementary Table S6.** NMI values of DGCSG and other baselines at different sequencing depths on neuron10k dataset.

| Sequencing depths | MBN | Contrastive-sc | nsDCC | scDC | scSMD | scDFC | scLEGA | scDFN | DGCSG |
|---|---|---|---|---|---|---|---|---|---|
| 35711159 reads (10%) | 0.6147 | 0.3864 | 0.2633 | <u>0.6402</u> | 0.1626 | — | 0.1567 | 0.4316 | **0.6536** |
| 107133478 reads (30%) | <u>0.6352</u> | 0.4875 | 0.2700 | 0.6020 | 0.4583 | — | 0.2110 | 0.5162 | **0.6782** |
| 178555797 reads (50%) | <u>0.6437</u> | 0.4939 | 0.3322 | 0.6255 | 0.4430 | — | 0.2611 | 0.5311 | **0.6622** |
| 249978116 reads (70%) | <u>0.6349</u> | 0.5118 | 0.3241 | 0.6166 | 0.4576 | — | 0.3197 | 0.5346 | **0.6640** |
| 357111595 reads (100%) | 0.6226 | 0.4913 | 0.3386 | <u>0.6319</u> | 0.4801 | — | 0.4595 | 0.5260 | **0.6615** |

'—' indicates missing values, due to out-of-memory running of scDFC on the neuron10k dataset.

**Supplementary Table S7.** ARI values of DGCSG and other baselines at different sequencing depths on heart10k dataset.

| Sequencing depths | MBN | Contrastive-sc | nsDCC | scDC | scSMD | scDFC | scLEGA | scDFN | DGCSG |
|---|---|---|---|---|---|---|---|---|---|
| 29043957 reads (10%) | 0.4126 | 0.1700 | 0.1572 | <u>0.7144</u> | 0.2636 | 0.4269 | 0.0495 | 0.3684 | **0.7534** |
| 87131871 reads (30%) | 0.4451 | 0.3149 | 0.1756 | <u>0.7289</u> | 0.3129 | 0.5250 | 0.1446 | 0.4155 | **0.7800** |
| 145219786 reads (50%) | 0.4991 | 0.4047 | 0.1695 | <u>0.7160</u> | 0.3302 | 0.1221 | 0.2154 | 0.4354 | **0.7915** |
| 203307700 reads (70%) | 0.5438 | 0.4194 | 0.1594 | <u>0.7277</u> | 0.3761 | 0.2231 | 0.2197 | 0.4482 | **0.7921** |
| 290439571 reads (100%) | 0.5657 | 0.4733 | 0.1704 | <u>0.7344</u> | 0.4453 | 0.2294 | 0.2326 | 0.4719 | **0.8012** |

**Supplementary Table S8.** ARI values of DGCSG and other baselines at different sequencing depths on neuron10k dataset.

| Sequencing depths | MBN | Contrastive-sc | nsDCC | scDC | scSMD | scDFC | scLEGA | scDFN | DGCSG |
|---|---|---|---|---|---|---|---|---|---|
| 35711159 reads (10%) | 0.4385 | 0.2132 | 0.1118 | 0.4405 | 0.0544 | — | 0.0341 | 0.2255 | **0.4953** |
| 107133478 reads (30%) | 0.4626 | 0.2777 | 0.1157 | 0.3904 | 0.1607 | — | 0.0288 | 0.3063 | **0.5349** |
| 178555797 reads (50%) | 0.4602 | 0.2927 | 0.1305 | 0.4194 | 0.2226 | — | 0.0839 | 0.3073 | **0.4904** |
| 249978116 reads (70%) | 0.4302 | 0.3005 | 0.1451 | 0.4163 | 0.1957 | — | 0.0786 | 0.3100 | **0.5056** |
| 357111595 reads (100%) | 0.4112 | 0.2849 | 0.1213 | 0.4237 | 0.2419 | — | 0.2286 | 0.2926 | **0.4764** |

'—' indicates missing values, due to out-of-memory running of scDFC on the neuron10k dataset.

**Supplementary Table S9.** NMI values of DGCSG and other baselines at different dropout rates on the simulated dataset.

| Dropout rates | MBN | Contrastive-sc | nsDCC | scDC | scSMD | scDFC | scLEGA | scDFN | DGCSG |
|---|---|---|---|---|---|---|---|---|---|
| 0% | 0.6192 | 1.0000 | 0.6121 | 1.0000 | 1.0000 | 0.8863 | 0.7370 | 0.6006 | **1.0000** |
| 23.1% | 0.5421 | 0.9786 | 0.3679 | **0.9978** | 0.9803 | 0.0152 | 0.0480 | 0.2075 | <u>0.9847</u> |
| 30.48% | 0.3678 | 0.9447 | 0.2132 | **0.9892** | <u>0.9792</u> | 0.0096 | 0.0920 | 0.0751 | 0.9443 |
| 38.89% | 0.3022 | <u>0.9239</u> | 0.1436 | 0.7547 | 0.8914 | 0.0082 | 0.0290 | 0.0492 | **0.9395** |
| 47.96% | 0.2999 | 0.7810 | 0.1264 | 0.3390 | <u>0.7874</u> | 0.0082 | 0.0200 | 0.0366 | **0.7920** |

**Supplementary Table S10.** ARI values of DGCSG and other baselines at different dropout rates on the simulated dataset.

| Dropout rates | MBN | Contrastive-sc | nsDCC | scDC | scSMD | scDFC | scLEGA | scDFN | DGCSG |
|---|---|---|---|---|---|---|---|---|---|
| 0% | 0.4993 | 1.0000 | 0.5067 | 1.0000 | 1.0000 | 0.7756 | 0.4740 | 0.4166 | **1.0000** |
| 23.1% | 0.3569 | 0.9865 | 0.3175 | **0.9988** | 0.9865 | 0.0074 | 0.0210 | 0.1396 | <u>0.9901</u> |
| 30.48% | 0.2961 | 0.9607 | 0.1724 | **0.9939** | <u>0.9864</u> | 0.0069 | 0.0580 | 0.0412 | 0.9611 |
| 38.89% | 0.2939 | <u>0.9471</u> | 0.1148 | 0.7051 | 0.7910 | 0.0038 | 0.0040 | 0.0299 | **0.9605** |
| 47.96% | 0.2880 | <u>0.8077</u> | 0.0959 | 0.2288 | 0.7868 | 0.0042 | 0.0080 | 0.0198 | **0.8334** |

**Supplementary Table S11.** Running time (seconds) of DGCSG and 16 baselines on 14 scRNA-seq datasets.

| Datasets | CCGC | MBN | SDCN | SCGC | Contrastive-sc | scNAME | nsDCC | scziDesk | scDC | scSMD | scGNN | scGAC | scTAG | scDFC | scLEGA | scDFN | DGCSG |
|---|---|---|---|---|---|---|---|---|---|---|---|---|---|---|---|---|---|
| Klein | 21 | 115 | 14 | 56 | 15 | 189 | 217 | 166 | 178 | 287 | 144 | 353 | 85 | 231 | 255 | 67 | 22 |
| Chung | 10 | 26 | 8 | 14 | 17 | 48 | 160 | 55 | 510 | 290 | 792 | 34 | 66 | 24 | 38 | 23 | 13 |
| Sun.1 | 14 | 52 | 10 | 26 | 25 | 125 | 16 | 95 | 43 | 307 | 41 | 111 | 62 | 97 | 77 | 30 | 11 |
| Sun.2 | 59 | 255 | 55 | 78 | 32 | 349 | 111 | 300 | 137 | 428 | 166 | 1192 | 131 | 628 | 314 | 171 | 59 |
| Sun.3 | 202 | 679 | 190 | 163 | 80 | 609 | 428 | 651 | 235 | 551 | 383 | 3605 | 268 | 2014 | 349 | 193 | 222 |
| Biase | 5 | 7 | 3 | 6 | 12 | 8 | 17 | 5 | 55 | 199 | 139 | 4 | 52 | 6 | 6 | 11 | 14 |
| Muraro | 27 | 103 | 34 | 38 | 22 | 159 | 158 | 128 | 1400 | 309 | 855 | 118 | 81 | 12 | 175 | 60 | 33 |
| Deng | 12 | 16 | 5 | 10 | 81 | 26 | 38 | 29 | 168 | 213 | 169 | 7 | 53 | 5 | 24 | 17 | 14 |
| Pollen | 26 | 15 | 4 | 10 | 81 | 24 | 49 | 29 | 226 | 220 | 195 | 7 | 54 | 6 | 25 | 19 | 7 |
| Darmanis | 24 | 21 | 5 | 12 | 83 | 37 | 128 | 40 | 266 | 218 | 242 | 13 | 57 | 6 | 35 | 23 | 10 |
| 10X_PBMC | 63 | 293 | 64 | 73 | 100 | 301 | 273 | 276 | 1876 | 379 | 1932 | 1060 | 142 | 113 | 419 | 132 | 68 |
| Mouse bladder | 36 | 140 | 38 | 55 | 106 | 205 | 233 | 207 | 1482 | 395 | 1412 | 171 | 102 | 22 | 290 | 78 | 40 |
| Habib | 595 | 1715 | 607 | 288 | 162 | 1446 | 1246 | 1651 | 8039 | 934 | 11327 | — | 497 | — | 502 | 510 | 517 |
| Zeisel | 38 | 155 | 47 | 182 | 89 | 208 | 386 | 170 | 2063 | 337 | 1375 | 436 | 97 | 154 | 264 | 161 | 42 |

'—' indicates missing values, due to out-of-memory running of scGAC and scDFC on the Habib dataset.

**Supplementary Table S12.** Significance test results of clustering performance between DGCSG and baselines in terms of ARI.

| Datasets | CCGC | MBN | SDCN | SCGC | Contrastive-sc | scNAME | nsDCC | scziDesk | scDC | scSMD | scGNN | scGAC | scTAG | scDFC | scLEGA | scDFN |
|---|---|---|---|---|---|---|---|---|---|---|---|---|---|---|---|---|
| Klein | 0.0000 | 0.0000 | 0.0000 | 0.0000 | 0.0000 | 0.0305 | 0.0000 | 0.0000 | 0.0000 | 0.0011 | 0.0000 | 0.0000 | 0.0000 | 0.0000 | 0.0000 | 0.0000 |
| Chung | 0.0000 | 0.0000 | 0.0000 | 0.0000 | 0.0059 | 0.0116 | 0.0000 | 0.0025 | 0.0000 | 0.0012 | 0.0000 | 0.0001 | 0.0000 | 0.0000 | 0.0000 | 0.0000 |
| Sun.1 | 0.0000 | 0.0000 | 0.0000 | 0.0000 | 0.0007 | 0.0087 | 0.0001 | 0.0036 | 0.0000 | 0.0105 | 0.0000 | 0.0001 | 0.0000 | 0.0000 | 0.0000 | 0.0000 |
| Sun.2 | 0.0000 | 0.0000 | 0.0000 | 0.0000 | 0.0001 | 0.0008 | 0.0005 | 0.0033 | 0.0000 | 0.0177 | 0.0000 | 0.0000 | 0.0000 | 0.0000 | 0.0000 | 0.0000 |
| Sun.3 | 0.0000 | 0.0000 | 0.0022 | 0.0000 | 0.0000 | 0.0104 | 0.0025 | 0.0000 | 0.0000 | 0.0000 | 0.0000 | 0.0061 | 0.0000 | 0.0000 | 0.0000 | 0.0000 |
| Muraro | 0.0000 | 0.0000 | 0.0000 | 0.0000 | 0.0040 | 0.0031 | 0.0018 | 0.0000 | 0.0000 | **0.1042** | 0.0000 | 0.0000 | 0.0000 | 0.0041 | 0.0000 | 0.0048 |
| Deng | 0.0496 | 0.0000 | 0.0000 | 0.0000 | 0.0259 | 0.0015 | 0.0000 | 0.0000 | 0.0000 | 0.0001 | 0.0000 | 0.0000 | 0.0000 | 0.0197 | 0.0050 | 0.0000 |
| Pollen | 0.0000 | 0.0000 | 0.0000 | 0.0000 | 0.0008 | 0.0032 | 0.0011 | 0.0000 | 0.0003 | 0.0004 | 0.0000 | 0.0000 | 0.0000 | 0.0137 | 0.0000 | 0.0026 |
| Darmanis | 0.0000 | 0.0000 | 0.0000 | 0.0000 | 0.0000 | 0.0000 | 0.0004 | 0.0000 | 0.0000 | 0.0002 | 0.0000 | 0.0010 | 0.0000 | 0.0005 | 0.0000 | 0.0000 |
| 10X_PBMC | 0.0000 | 0.0018 | 0.0000 | 0.0000 | 0.0000 | 0.0001 | 0.0008 | 0.0000 | 0.0089 | 0.0000 | 0.0000 | 0.0034 | 0.0000 | 0.0000 | 0.0000 | 0.0000 |
| Mouse bladder | 0.0000 | 0.0000 | 0.0000 | 0.0000 | 0.0000 | 0.0040 | 0.0000 | 0.0000 | 0.0014 | 0.0018 | 0.0000 | 0.0001 | 0.0000 | 0.0000 | 0.0000 | 0.0003 |
| Habib | 0.0002 | 0.0000 | 0.0107 | 0.0000 | 0.0000 | 0.0003 | 0.0009 | 0.0052 | 0.0004 | **0.4218** | 0.0000 | — | 0.0000 | — | 0.0000 | **0.0000** |
| Zeisel | 0.0000 | 0.0000 | 0.0000 | 0.0000 | 0.0000 | 0.0004 | 0.0001 | 0.0000 | 0.0000 | 0.0064 | 0.0000 | 0.0001 | 0.0000 | 0.0000 | 0.0000 | 0.0217 |

'—' indicates missing values, due to out-of-memory running of scGAC and scDFC on the Habib dataset.

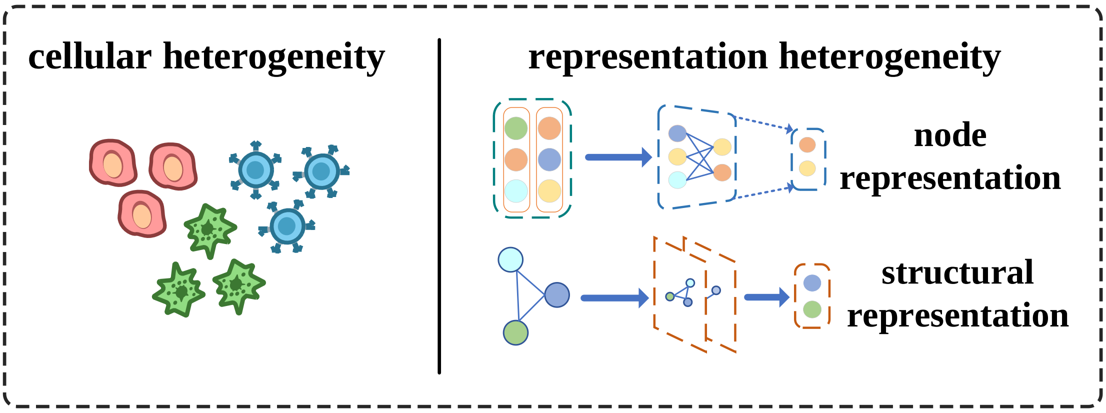


**Supplementary Fig. S1.** The difference between cellular heterogeneity and representation heterogeneity. Cellular heterogeneity is a biological concept that refers to differences in gene expression, morphology, and function of cells. The heterogeneity between cell nodes and structural information is due to the differences in representation sources and information expression patterns, which leads to differences between the learned cell node representation and structural representation.

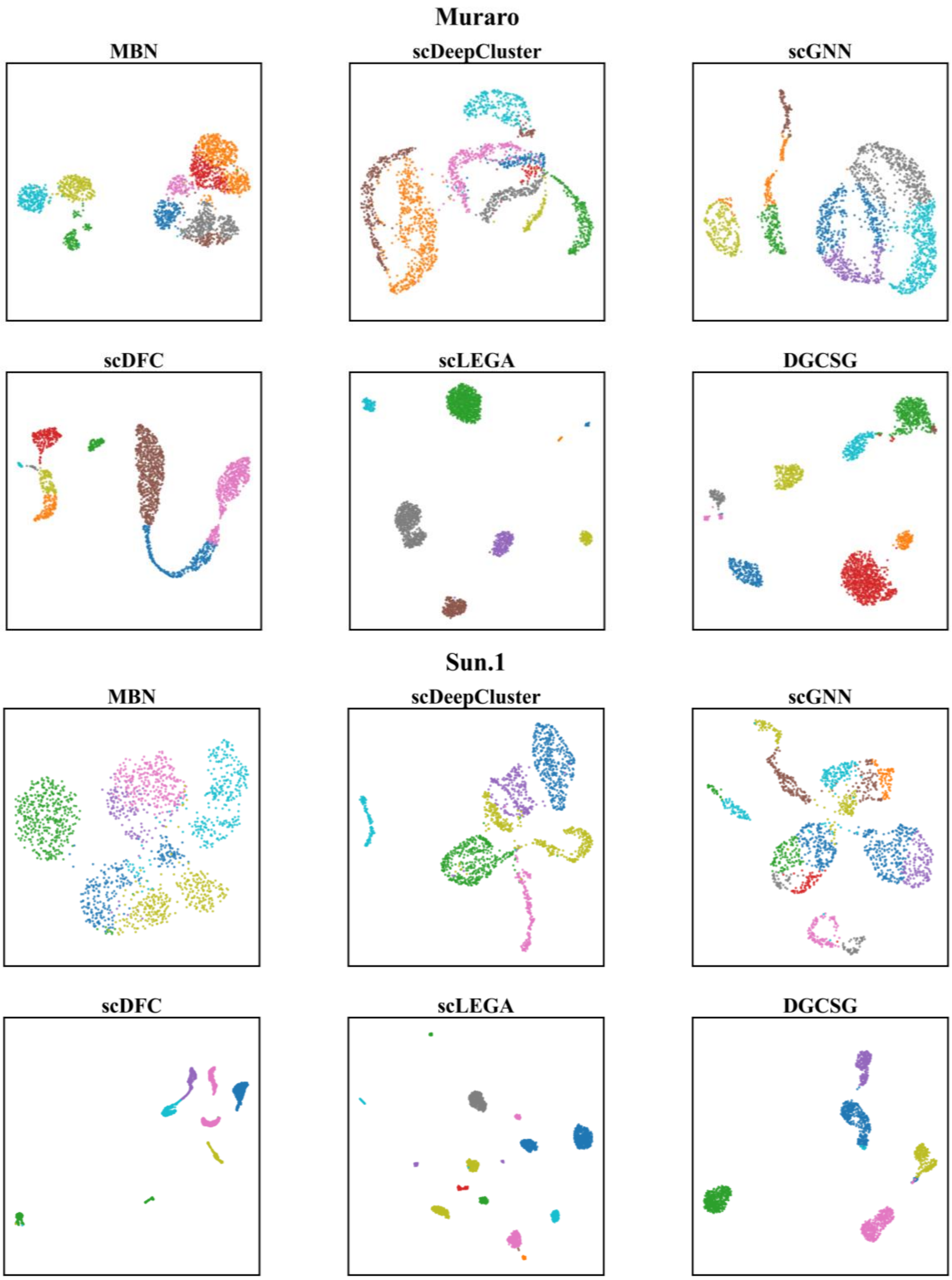


**Supplementary Fig. S2.** Visualization of cell representations for the Muraro and Sun.1 datasets. The colors represent the clustering labels of each approach.

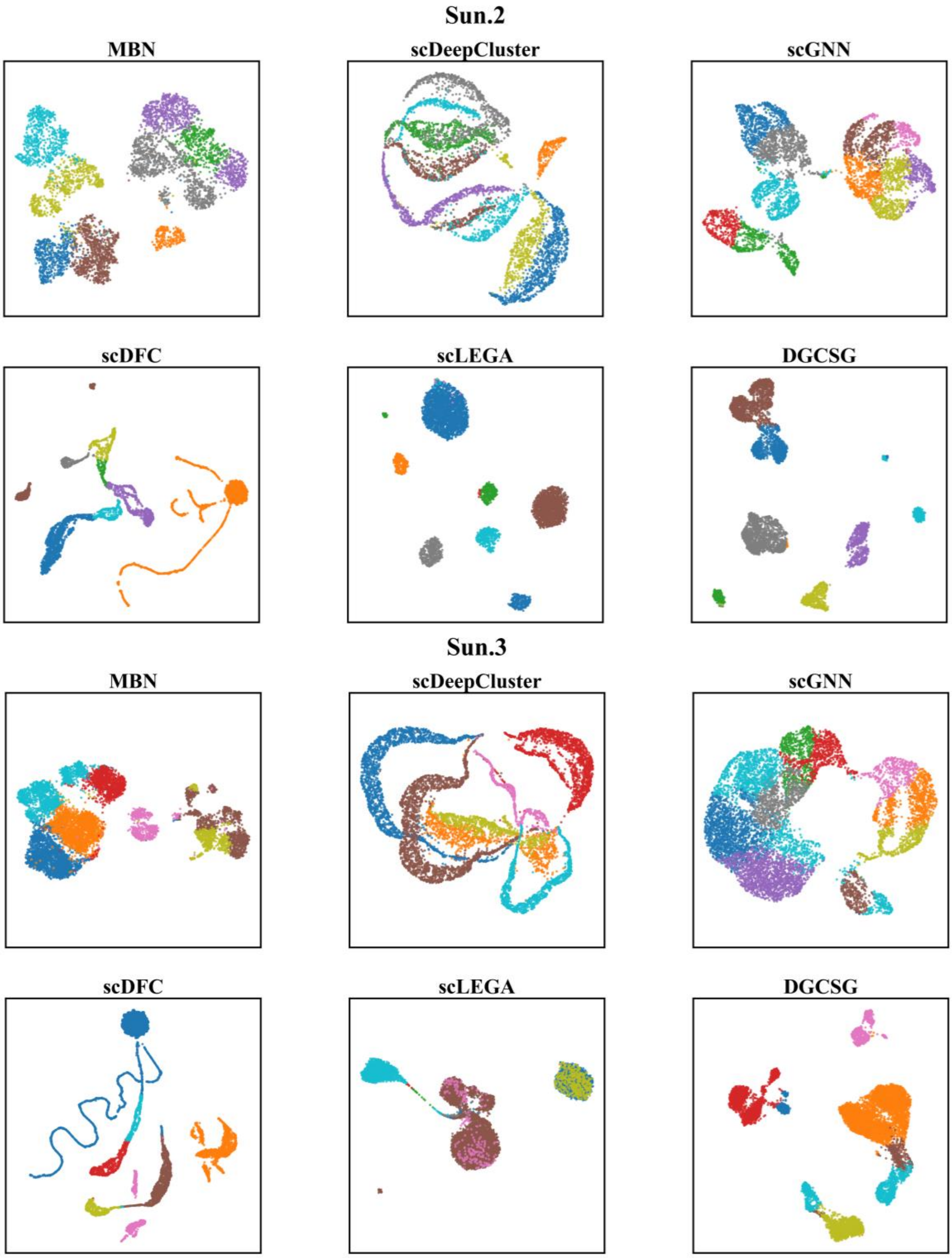


**Supplementary Fig. S3.** Visualization of cell representations for the Sun.2 and Sun.3 datasets. The colors represent the clustering labels of each approach.

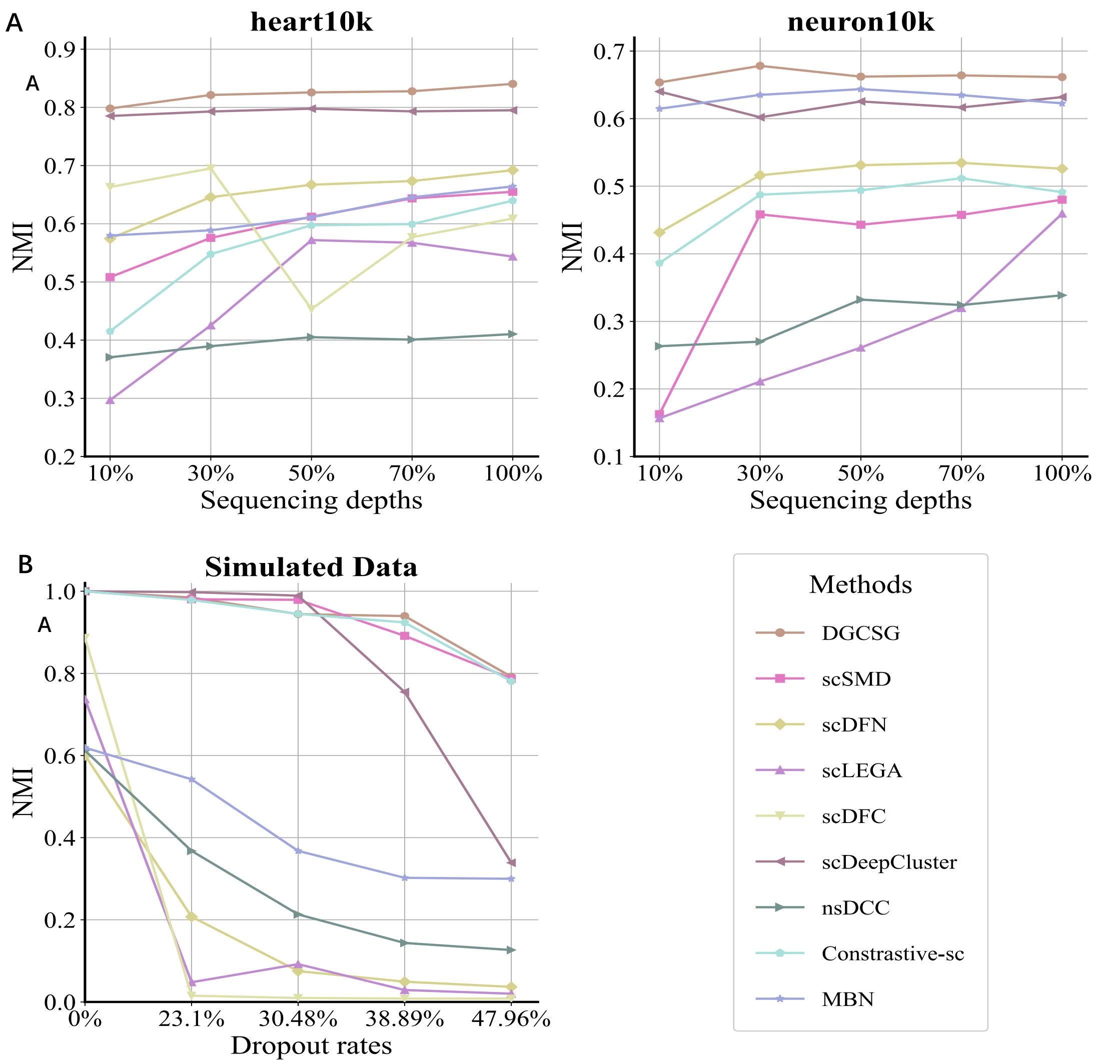


**Supplementary Fig. S4.** NMI values of DGCSG and other baselines at different conditions. (A) NMI values of DGCSG and other baselines at different sequencing depths on heart10k dataset and neuron10k dataset. Note that scDFC is not included in the figure due to out-of-memory error. (B) NMI values of DGCSG and other baselines at different dropout rates on the simulated dataset.

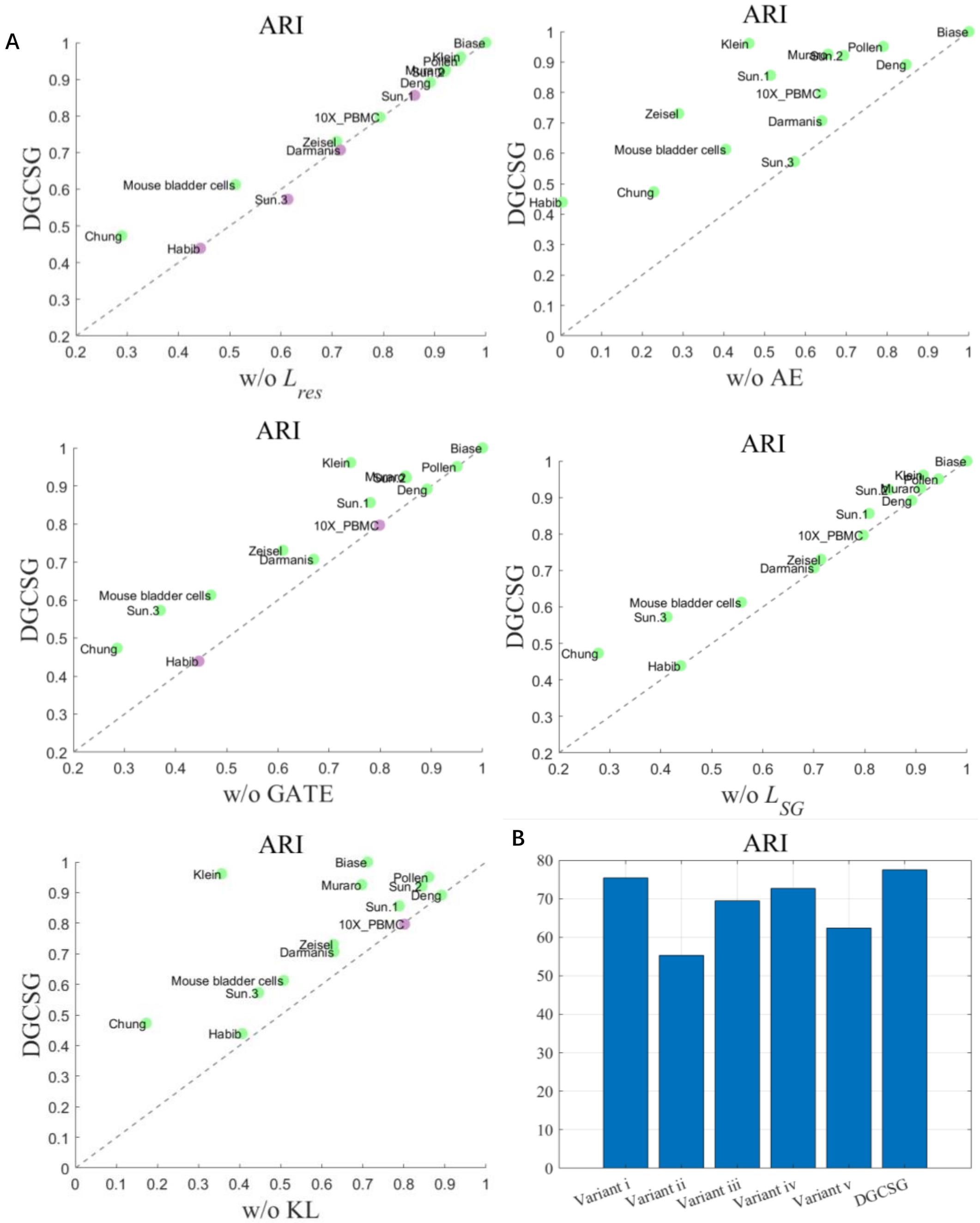


**Supplementary Fig. S5.** Ablation study for DGCSG. (A) Comparison of ARI metrics between DGCSG and the 5 variants on 14 datasets. (B) Comparison of the average ARI metrics between DGCSG and the 5 variants on 14 datasets, where variant i is w/o $\mathcal{L}_{res}$ , variant ii is w/o AE, variant iii is w/o GATE, variant iv is w/o $\mathcal{L}_{SG}$ , and variant v is w/o KL.

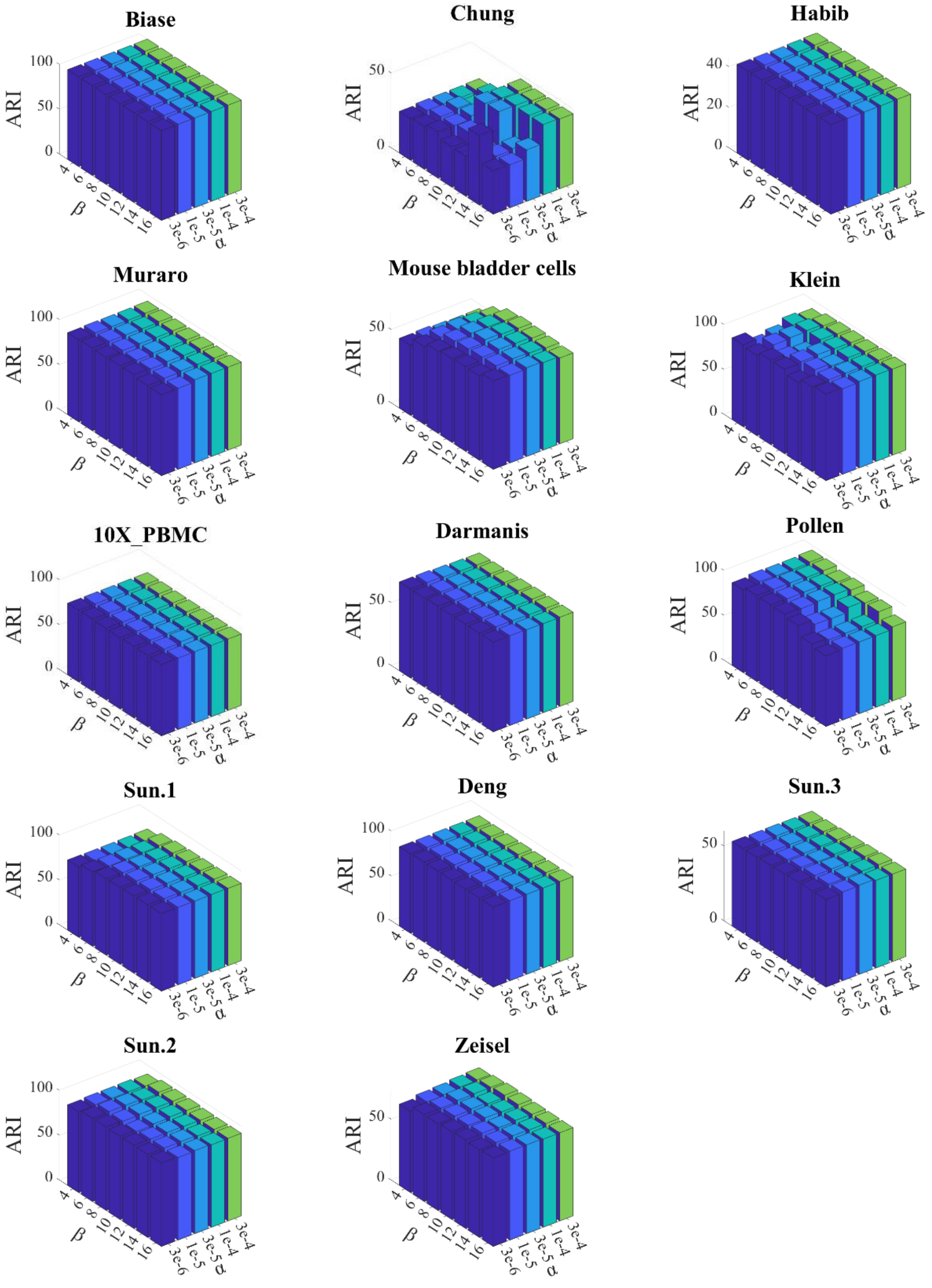


**Supplementary Fig. S6.** Sensitivity analysis of $\alpha$ and $\beta$ for DGCSG on 14 datasets.

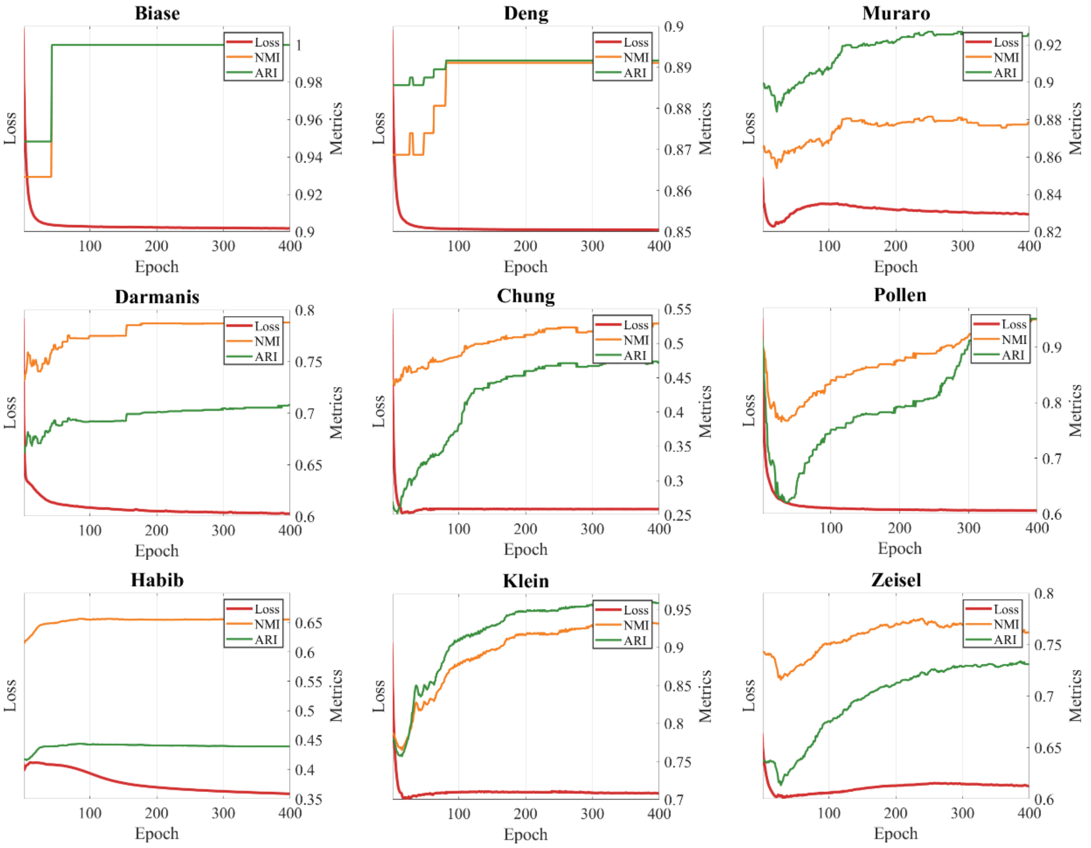

**Supplementary Fig. S7.** Convergence analysis of DGCSG on 9 datasets.

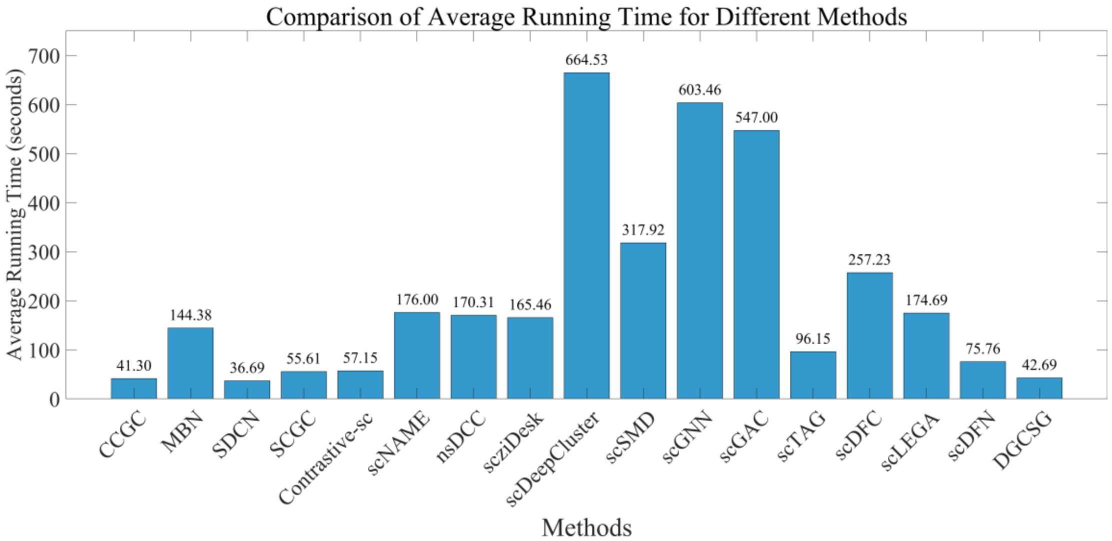


**Supplementary Fig. S8.** Average running time (seconds) of DGCSG and 16 baselines on 13 datasets except Habib. Since scGAC and scDFC failed to run on the Habib dataset due to out-of-memory, the Habib dataset is excluded when calculating the average running time.

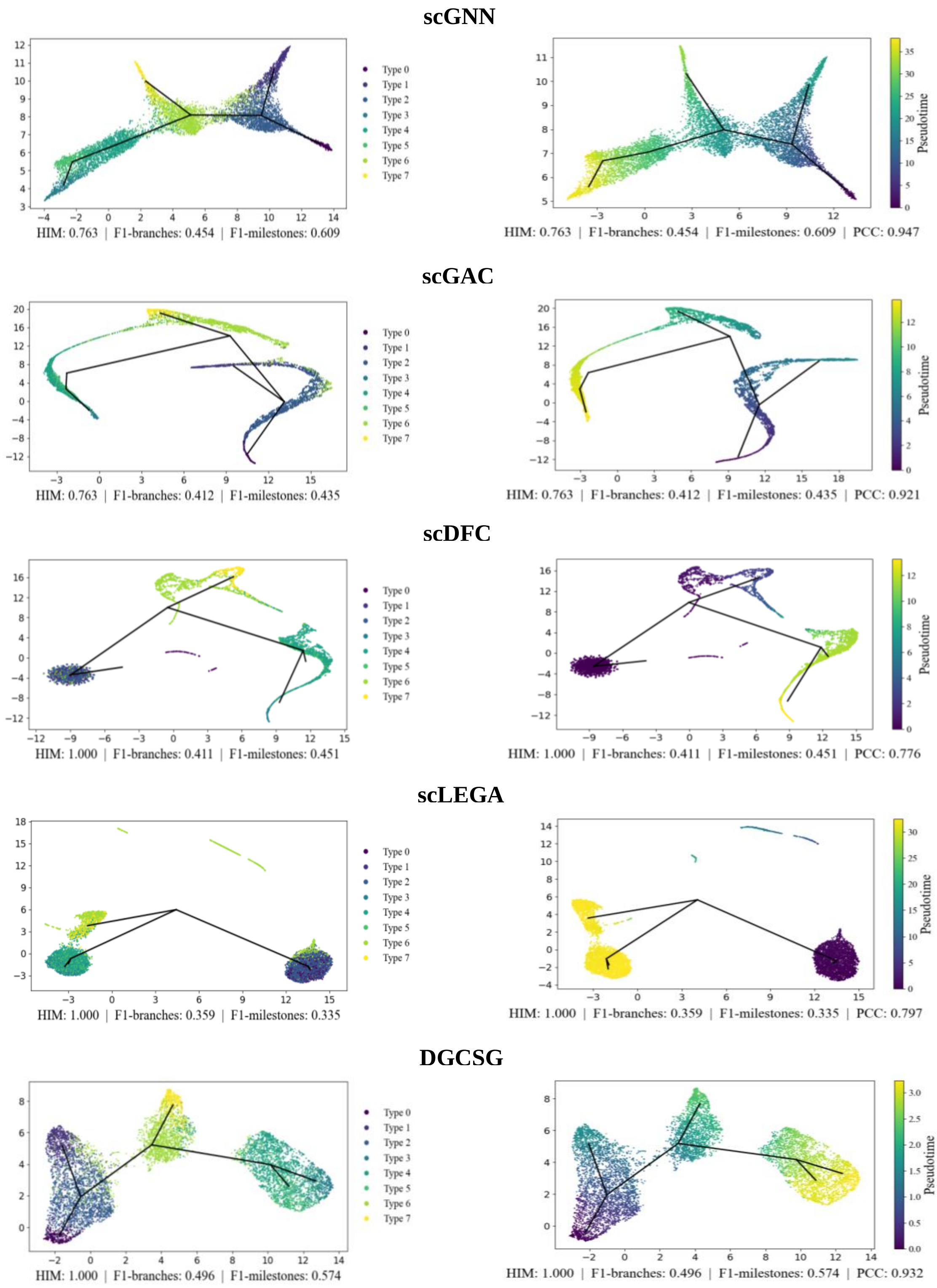


**Supplementary Fig. S9.** Visualization of cell trajectory inference using representations learned by DGCSG and 4 baselines. The left side color coded according to the cell labels, and the right side color coded according to the pseudo-time.

**References**


[1] Klein A M, Mazutis L, Akartuna I, *et al.* (2015) Droplet barcoding for single-cell transcriptomics applied to embryonic stem cells. *Cell***, 161:** 1187-1201.

[2] Chung W, Eum H H, Lee H O, *et al.* (2017) Single-cell RNA-seq enables comprehensive tumour and immune cell profiling in primary breast cancer. *Nat. Commun.***, 8:** 15081.

[3] Sun Z, Chen L, Xin H, *et al.* (2019) A Bayesian mixture model for clustering droplet-based single-cell transcriptomic data from population studies. *Nat. Commun.***, 10:** 1649.

[4] Biase F H, Cao X, Zhong S. (2014) Cell fate inclination within 2-cell and 4-cell mouse embryos revealed by single-cell RNA sequencing. *Genome Res.***, 24:** 1787-1796.

[5] Muraro M J, Dharmadhikari G, Grün D, *et al.* (2016) A single-cell transcriptome atlas of the human pancreas. *Cell Syst.***, 3:** 385-394.

[6] Deng Q, Ramsköld D, Reinius B, *et al.* (2014) Single-cell RNA-seq reveals dynamic, random monoallelic gene expression in mammalian cells. *Science***, 343:** 193-196.

[7] Pollen A A, Nowakowski T J, Shuga J, *et al.* (2014) Low-coverage single-cell mRNA sequencing reveals cellular heterogeneity and activated signaling pathways in developing cerebral cortex. *Nat. Biotechnol.***, 32:** 1053-1058.

[8] Darmanis S, Sloan S A, Zhang Y, *et al.* (2015) A survey of human brain transcriptome diversity at the single cell level. *Proc. Natl. Acad. Sci.***, 112:** 7285-7290.

[9] Zheng G X Y, Terry J M, Belgrader P, *et al.* (2017) Massively parallel digital transcriptional profiling of single cells. *Nat. Commun.***, 8:** 14049.

[10] Han X, Wang R, Zhou Y, *et al.* (2018) Mapping the mouse cell atlas by microwell-seq. *Cell***, 172:** 1091-1107.

[11] Habib N, Avraham-Davidi I, Basu A, *et al.* (2017) Massively parallel single-nucleus RNA-seq with DroNc-seq. *Nat. Methods***, 14:** 955-958.

[12] Zeisel A, Muñoz-Manchado A B, Codeluppi S, *et al.* (2015) Cell types in the mouse cortex and hippocampus revealed by single-cell RNA-seq. *Science***, 347:** 1138-1142.
